# A new approach to sustainable logistic processes with q-rung orthopair fuzzy soft information aggregation

**DOI:** 10.7717/peerj-cs.1527

**Published:** 2023-08-28

**Authors:** Muhammad Riaz, Hafiz Muhammad Athar Farid, Ayesha Razzaq, Vladimir Simic

**Affiliations:** 1 Department of Mathematics, University of the Punjab, Lahore, Punjab, Pakistan; 2 Faculty of Transport and Traffic Engineering, University of Belgrade, Belgrade, Serbia; 3 College of Engineering, Department of Industrial Engineering and Management, Yuan Ze University, Taoyuan, Taiwan

**Keywords:** q-rung orthopair fuzzy soft set, Aggregation operators, Sustainable logistic processes, Decison-making

## Abstract

In recent years, as corporate consciousness of environmental preservation and sustainable growth has increased, the importance of sustainability marketing in the logistic process has grown. Both academics and business have increased their focus on sustainable logistics procedures. As the body of literature expands, expanding the field’s knowledge requires establishing new avenues by analyzing past research critically and identifying future prospects. The concept of “q-rung orthopair fuzzy soft set” (q-ROFSS) is a new hybrid model of a q-rung orthopair fuzzy set (q-ROFS) and soft set (SS). A q-ROFSS is a novel approach to address uncertain information in terms of generalized membership grades in a broader space. The basic alluring characteristic of q-ROFS is that they provide a broader space for membership and non-membership grades whereas SS is a robust approach to address uncertain information. These models play a vital role in various fields such as decision analysis, information analysis, computational intelligence, and artificial intelligence. The main objective of this article is to construct new aggregation operators (AOs) named “q-rung orthopair fuzzy soft prioritized weighted averaging” (q-ROFSPWA) operator and “q-rung orthopair fuzzy soft prioritized weighted geometric” (q-ROFSPWG) operator for the fusion of a group of q-rung orthopair fuzzy soft numbers and to tackle complexities and difficulties in existing operators. These AOs provide more effective information fusion tools for uncertain multi-attribute decision-making problems. Additionally, it was shown that the proposed AOs have a higher power of discriminating and are less sensitive to noise when it comes to evaluating the performances of sustainable logistic providers.

## Introduction

Sustainable logistics processes are becoming increasingly important in today’s world. The transportation of goods and materials is one of the largest contributors to global greenhouse gas emissions, which are a major cause of climate change. In addition, the logistics sector has a significant impact on the environment through its use of energy and natural resources. The importance of sustainable logistics processes lies in their ability to reduce the environmental impact of transportation and logistics activities. Sustainable logistics can help to reduce greenhouse gas emissions by using more fuel-efficient vehicles, optimizing transportation routes, and reducing the distance traveled. It can also help to reduce the consumption of natural resources and minimize waste through recycling and waste reduction initiatives. Sustainable logistics also has economic benefits. By improving the efficiency of transportation and logistics, companies can reduce their operating costs and improve their bottom line. They can also improve their reputation and brand image by demonstrating their commitment to sustainability. The scope of sustainable logistics is broad and encompasses a wide range of activities. It includes the planning and management of transportation and logistics operations, as well as the design and development of transportation infrastructure. It also includes the use of sustainable fuels and technologies, such as electric and hybrid vehicles, as well as the optimization of transportation networks to minimize environmental impact. In order to implement sustainable logistics processes, companies need to adopt a holistic approach that takes into account the entire supply chain. This involves collaboration with suppliers and customers to optimize transportation routes, reduce waste, and improve efficiency. It also requires the use of data analytics and technology to track and monitor transportation and logistics activities and identify opportunities for improvement. Sustainable logistics processes are essential for reducing the environmental impact of transportation and logistics activities, improving economic performance, and demonstrating a commitment to sustainability. By adopting a holistic approach and collaborating with suppliers and customers, companies can achieve these benefits while also reducing their carbon footprint and contributing to a more sustainable future. Multi-attribute decision-making (MADM) is an important tool for sustainable logistics processes. In sustainable logistics, decisions need to be made considering various attributes such as cost, environmental impact, social responsibility, and operational efficiency. MADM can help decision-makers to evaluate and compare different alternatives based on multiple criteria, and select the most suitable option that meets the objectives of sustainable logistics. One of the key advantages of MADM is that it allows decision-makers to consider multiple attributes simultaneously, rather than just focusing on one or two. This helps to ensure that decisions are based on a comprehensive and balanced analysis of all relevant factors. For example, when evaluating transportation alternatives, MADM can help to balance the trade-off between cost and environmental impact, by considering factors such as fuel efficiency, emissions, and distance traveled. MADM also provides a structured and transparent decision-making process. This is particularly important for sustainable logistics, where decisions need to be made with the involvement of multiple stakeholders, including customers, suppliers, and regulators. MADM can help to ensure that all relevant stakeholders are involved in the decision-making process and that decisions are made transparently and objectively. Furthermore, MADM can help decision-makers to prioritize their sustainability goals. Sustainable logistics involves balancing different objectives, such as reducing costs, improving efficiency, and minimizing environmental impact. MADM can help to rank these objectives and determine which ones are most important, based on the specific context and goals of the logistics operation. Finally, MADM can help decision-makers to identify and evaluate trade-offs between different attributes. Sustainable logistics often involves making trade-offs between different goals, such as reducing emissions and increasing operational efficiency. MADM can help decision-makers to identify the tradeoffs between these different attributes, and evaluate the impact of different alternatives on each attribute. MADM is an important tool for sustainable logistics processes. It allows decision-makers to consider multiple attributes simultaneously, provides a structured and transparent decision-making process, helps to prioritize sustainability goals, and enables decision-makers to identify and evaluate trade-offs between different attributes. By using MADM, sustainable logistics can be achieved while also improving economic performance and demonstrating a commitment to sustainability.

### Main endowments and objectives

The main endowments and the goals of this article are given below:

- To establish the prioritized weighted averaging operators and prioritized weighted geometric operators under q-ROFS context, which deals with the prioritization connection in the information. Consequently, to handle such information, q-ROFSPWA and q-ROFSPWG operators are effectively presented.
- To present definite some basic properties of the proposed operators. Some fundamental properties including, comprising, monotonicity and boundedness are presented and examined with suitable development.
- Based on the q-ROFSPWA operator and q-ROFSPWG operator, a MADM algorithm is established to resolve some decision-making numerical problems.
- A fully developed numerical example is provided to validate the significance of the proposed operators.
- The importance of the proposed technique is emphasized through a comparison with existing approaches.
- The q-ROFSS is more suitable for examining decision-making problems than SS and q-ROFS.
- The evaluation of the finest alternative is a very complicated MADM problem in a q-ROFS and SS environment and has many indefinite components. In the existing MADM methods, evaluation data is simply illustrated by SS and q-ROFNs which may inspire data distortion. As a result, a more extensive model is required to elucidate the existence of universal components.
- The q-ROFSPWA and q-ROFSPWG operators when apply to different MADM difficulties based on the q-ROFS environment, increase the accuracy of the decision results.
- The q-ROFSPWA and q-ROFSPWG operators are very straightforward and brief method for the assessment of a single choice.
- Proposed operators address the shortcomings and constraints of existing operators by being more general and performing well with data other than q-ROFS.

The following are the main features of this article:

- The theory of q-ROF aggregation operators is expanded to q-ROFS operators, with some basic results associated with them.
- A method for dealing with difficult problems using q-ROFS data is provided. The MADM problem helps the proposed algorithm.
- Various parameter choices and their effects on decision-making outcomes are reviewed.
- The comparative analysis demonstrates the efficacy of these operators.

The structure of this article is given as: In “Literature Review” some literature review has given and in “Fundamental Notions” some basic ideas including q-ROFS, some basic operations, score function and accuracy function of q-ROFNs and q-ROFSS are presented which are useful to understand the proposed operators. “q-ROFS Prioritized Weighted Aggregation Operators” presents the prioritized operators like the q-ROFSPWA operator and q-ROFSPWG operator, and also presents some beneficial characteristics of proposed operators. In “Proposed MADM Approach”, an algorithm of the proposed work is provided. “Case Study” involves a numerical example and authenticity, sensitivity, and symmetrical analysis. Eventually, the conclusion is provided in “Conclusion”.

## Literature review

An essential challenge in the MADM process is expressing approximate values of attributes more efficiently and precisely. The precise parameterized values of attributes are useful to address several complexities in MADM challenges. The decision experts estimate the advantages, characteristics and limitations of universal elements, goods, and alternatives. MADM is a predesign procedure for the selection of the best option among multiple choices. To handle these challenges, Zadeh (1965) presented a conspicuous idea, namely fuzzy set (FS) which made the best gyration in various fields. In an FS, membership values (MVs) between 0 and 1 are assigned to each alternative. Nevertheless, in some real-life problems decision experts gives their evaluations in terms of MV and negative membership value (NMV). Accordingly, Atanassov (1986) established the generalization of FS, namely “intuitionistic fuzzy set” (IFS) which contained MV and NMV functions that expressed satisfactory and unsatisfactory levels, respectively. As a result, it is a particularly useful tool for expressing complicated fuzzy data. Xu (2007), Xu & Yager (2006), Xu & Xia (2011) presented induced generalized, weighted averaging and weighted geometric operators based on intuitionistic fuzzy numbers (IFNs). Yager (2013) presented a “Pythagorean fuzzy set” (PFS) with a positive membership value (PMV) and negative membership value (NMV) that fulfilled the criteria that the square of the sum of its PMV and NMV is less than or equal to one. Based on the of extenuating TOPSIS technique (Hadi-Vencheh & Mirjaberi, 2014), Zhang (2016) designed a TOPSIS technique for MADM problems, containing PFS information. Simultaneously, Peng and Yang presented PFS sets (PFSSs) (Peng et al., 2015) and Pythagorean fuzzy linguistic sets (PFLSs) (Peng & Yang, 2016), respectively, inspirited by SS theory (Molodtsov, 1999) and linguistic set (LS) theory (Zadeh, 1975a, 1975b, 1975c). Gou, Xu & Ren (2016) established different Pythagorean fuzzy functions and thoroughly explored their important features such as differentiability, continuity and drivability. Peng & Yang (2015) introduced division and subtraction operations, as well as the Pythagorean fuzzy inferiority and superiority ranking system for solving MADM problems with PFNs. Following that, Beliakov & James (2014) studied on how to interpret the word “averaging” in the context of PFNs.

Despite this, Yager (2016) first developed the q-ROFSs to epitomize the decision information, in which the aggregate sum of the qth power of PMV and NMV is below or equal to 1, $0 \le {\mu ^{\it q}} + {\nu ^{\it q}} \le 1,(q \ge 1)$. It should be noted that as ‘q’ raises, so does the space of admissible orthopairs, and more orthopairs meet the boundary restriction. Using q-ROFSs, we may represent a broader space of fuzzy data. In other words, we may keep changing the ‘q’ value to define the data representation range, making q-ROFS more flexible and appropriate for uncertainty. Liu & Wang (2018), Wei, Gao & Wei (2018) and Liu & Liu (2018), Liu, Chen & Wang (2018), Liu, Chen & Wang (2018) proposed some novel q-ROF AOs for aggregating the q-ROF information. Based on some score functions, Peng, Dai & Garg (2018) introduced new AOs and exponential operations for q-ROFS and used them for teaching system selection. Du (2018) proposed some Minkowski type distance measures for q-ROFSs like, Chebyshev, Eucledian and Hamming distances as well as analyzed their implications in MADM challenges. Liu, Liu & Liang (2018) introduced a new MADM technique for contending with diverse connection in parameters and uncertain weight information of attributes in a q-ROF context. In a q-ROF framework, Yager, Alajlan & Bazi (2018) studied the ideas of certainty, feasibility as well as belief and plausibility. Pinar & Boran (2020) evaluated and explored another distance measure for q-ROF values in detail. Using the proposed distance measure, they examined the supplier selection problem using the TOPSIS and ELECTRE techniques individually. Based on Dombi and Archimedean operations, Saha, Dutta & Kar (2021) developed some novel hesitant fuzzy weighted aggregation operations and their applications. Based on Aczel-Alsina operations, Senapati et al. (2022) developed interval-valued intutionistic fuzzy AOs. Mahmood et al. (2019) established a novel technique, based on spherical fuzzy sets. Jana, Muhiuddin & Pal (2019) provided some Dombi aggregation operators for q-ROFNs and their different applications in MADM.

Yang et al. (2021) explored the numerous heterogeneous relationships between membership functions and criteria. The real world is just too complicated for our direct comprehension. We develop models that are simplified versions of real situations. However, these mathematical models are too complex to find accurate solutions. The ambiguity of data when modelling challenges in physics, engineering, computer science, social sciences, economics, medical science ad many other domains finds traditional methodologies ineffective. These can be related to the uncertainties of natural environmental phenomena, human awareness of the real world, or the constraints of the measurement tools applied. For example, ambiguity or uncertainty in the boundary between urban and rural regions or between states, or making judgments in a machine-based environment utilizing database information, or the precise population growth in a country’s rural area. The above-mentioned theories can be regarded as tools for coping with uncertainty, but each of these ideas has its own set of difficulties. The reason for these challenges may be the insufficiency of the parameterized description of elements, as highlighted by Molodtsov (1999). He developed the notion of SS theory as a new mathematical tool to handle. Maji, Roy & Biswas (2002) demonstrated the first practical use of SS in decision-making situations. It is based on the rough set theory of knowledge reduction. In 2003, Maji, Biswas & Roy (2003) established and explored many fundamental concepts of SS theory. Chen et al. (2005) and Pei & Miao (2005) amended the work of Maji, Roy & Biswas (2002), Maji, Biswas & Roy (2003). Maji, Biswas & Roy (2001a) introduced a fuzzy soft set (FSS), a hybrid of soft set and fuzzy set, that had various applications. Maji, Biswas & Roy (2001b) developed an extension of FSS named as intuitionistic FSS (IFSS). Hamid, Riaz & Afzal (2020) developed a q-ROF soft set (q-ROFSS). By utilizing averaging operators, Hussain et al. (2020) developed MADM approaches on q-ROFSS. The concept of score functions associated with generalized orthopair fuzzy membership grades, along with their practical applications, was suggested by Feng et al. (2022). Sitara, Akram & Riaz (2021) developed graph structures of q-ROFSs and a decision-making approach utilizing these structures.

q-ROFSs have been used for personal mobility in the metaverse with driverless cars, socially responsible rehabilitation of mining sites, and floating offshore wind farm site selection in Norway (Deveci et al., 2022; Deveci, Gokasar & Brito-Parada, 2022; Deveci et al., 2022). Farid & Riaz (2022), Riaz et al. (2020) proposed some AOs with applications to green supplier selection and Liu & Wang (2018) introduced interval-valued intuitionistic fuzzy Schweizer–Sklar power AOs with supplier selection applications. Liu et al. (2022) used the techniques of the operational science for green supplier selection with cross-entropy and Archimedean AOs. Zulqarnain et al. (2021) introduced some AOs for Pythagorean fuzzy soft sets with their application to green supplier chain management. Zhang, Wei & Chen (2021) and Qiyas & Abdullah (2022) gave some brilliant decision-making method for supplier selection. Wei et al. (2022), Pinar, Babak Daneshvar & Özdemir (2021) and Krishankumar et al. (2021) proposed AOs for different extensions of fuzzy sets and their applications towards vendor selection. Some extensive work related to MADM can be seen in Wan, Jiuying & Deyan (2015), Dong & Wan (2016), Puška et al. (2023), Rahman et al. (2023), Riaz & Farid (2023), Kausar, Riaz & Farid (2023). Finally, some existing AOs related to different extensions of FSs are given in Table 1.

**Table 1:** Some existing aggregation operators.

Authors	Aggregation operators
Wei, Gao & Wei (2018)	q-ROF Heronian mean aggregation operators
Senapati et al. (2022)	Interval-valued intutionistic fuzzy Aczel-Alsina AOs
Wang & Li (2020)	Pythagorean fuzzy interaction power Bonferroni mean AOs
Wei (2017)	Pythagorean fuzzy interaction weighted AOs
Jana, Muhiuddin & Pal (2019)	q-ROF Dombi aggregation operators
Farid & Riaz (2021)	q-ROF Einstein interactive geometric AOs
Riaz et al. (2021)	q-ROF interactive AOs
Garg (2016)	Intuitionistic fuzzy Hamacher interactive weighting AOs
Garg & Arora (2018)	Prioritized intuitionistic fuzzy soft interactive AOs

According to the preceding analysis, the majority of existing q-ROFS aggregate relies on the algebraic product and algebraic sum of q-ROFSSs to carry out the aggregation process, which does not consider the interdependence among the multi factors. It is necessary to construct some underlying operators that can handle MADM problems in various situations of information combinations. Furthermore, the extension of q-ROFSs is the generalized version for dealing with any embeddings. In this regard, there is a considerable opportunity to exercise a different perspective of prioritized aggregation operators since the q-ROFSSs deliver ambiguous information in more productive ways.

## Fundamental notions

Some basic notions of q-ROFS, “score function” (SF), “accuracy functions” (AF) and some laws of q-ROFNs are presented in this section.

**Definition 3.1** Yager (2013): *A q-ROFS* ${\mathcal O}$ *in* ${ \mathcal 2}$ *is determined as*

$${\mathcal O} = \{ \langle \complement,{\mu _{\mathcal O}}(\complement),{\nu _{\mathcal O}}(\complement)\rangle :\complement \in {\mathcal 2}\}$$

*where* $q \ge 1$. ${\mu _{\mathcal O}}(\complement),{\nu _{\mathcal O}}(\complement)$ *represents the MV and NMV of the universal elements* $\complement \in {\mathcal 2}$*,* *we have*

$$0 \le \mu _{\mathcal O}^{\it q}(\complement) + \nu _{\mathcal O}^{\it q}(\complement) \le 1.$$

*Moreover,* ${\pi _{\mathcal O}}(\complement) = {(1 - \mu _{\mathcal O}^{\it q}(\complement) - \nu _{\mathcal O}^{\it q}(\complement))^{1/{\it q}}}$ *is said to be the degree of indeterminacy* $\complement$ *to* ${\mathcal O}$.

The following operational laws are presented by Liu & Wang (2018) for q-ROFN information.

**Definition 3.2** Liu & Wang (2018): Let ${{\mathcal N}^{\beth}}_1 = \langle {\mu _1},{\nu _1}\rangle$ and ${{\mathcal N}^{\beth}}_2 = \langle {\mu _2},{\nu _2}\rangle$ be q-ROFNs. Then

(1) ${{{\bar{\mathcal N}}^{{\beth}}}_1} = \langle {\nu _1},{\mu _1}\rangle$

(2) ${{\mathcal N}^{\beth}}_1 \vee {{\mathcal N}^{\beth}}_2 = \langle {\mathrm{max}}\{ {\mu _1},{\nu _1}\} ,{\mathrm{min}}\{ {\mu _2},{\nu _2}\} \rangle$

(3) ${{\mathcal N}^{\beth}}_1 \wedge {{\mathcal N}^{\beth}}_2 = \langle {\mathrm{min}}\{ {\mu _1},{\nu _1}\} ,{\mathrm{max}}\{ {\mu _2},{\nu _2}\} \rangle$

(4) ${{\mathcal N}^{\beth}}_1 \oplus {{\mathcal N}^{\beth}}_2 = \langle {(\mu _1^{\it q} + \mu _2^{\it q} - \mu _1^{\it q}\mu _2^{\it q})^{1/q}},{\nu _1}{\nu _2}\rangle$

(5) ${{\mathcal N}^{\beth}}_1 \otimes {{\mathcal N}^{\beth}}_2 = \langle {\mu _1}{\mu _2},{(\nu _1^{\it q} + \nu _2^{\it q} - \nu _1^{\it q}\nu _2^{\it q})^{1/q}}\rangle$

(6) $\sigma {{\mathcal N}^{\beth}}_1 = \langle {(1 - {(1 - \mu _1^{\it q})^\sigma })^{1/q}},\nu _1^\sigma \rangle$

(7) ${{\mathcal N}^{\beth}}_1^\sigma = \langle \mu _1^\sigma ,{(1 - {(1 - \nu _1^{\it q})^\sigma })^{1/q}}\rangle$

**Definition 3.3** Liu & Wang (2018): Consider $\tilde \partial = \langle \mu ,\nu \rangle$ is a q-ROFN, the SF $({\mathcal L})$ of $\widetilde {\eth}$ is represented as,

$${\mathcal S}(\tilde \Omega ) = {\mu ^{\it q}} - {\nu ^{\it q}}$$

${\mathcal S}(\tilde \Omega ) \in [ - 1,1]$. The SF of a q-ROFNs determines its classification. However, SF is not useful in a number of cases involving q-ROFNs.

**Definition 3.4** Liu & Wang (2018): Suppose $\widetilde {\eth} = \langle \mu ,\nu \rangle$ is a q-ROFN, the AF ${\mathcal A}$ of $\widetilde {\eth}$ is determined as

$${\mathcal A}(\tilde \Omega ) = {\mu ^{\it q}} + {\nu ^{\it q}}$$

${\mathcal A}(\tilde \Omega ) \in [0,1]$. The high preference of $\tilde \Omega$ is determined by the high accuracy degree ${\mathcal A}(\tilde \Omega )$.

**Definition 3.5** Liu & Wang (2018): Suppose that ${{\mathcal N}^{\beth}}_k = \langle {\mu _k},{\nu _k}\rangle$ be a agglomeration of q-ROFNs, and q-ROFWA $:{\Lambda ^n} \to \Lambda$, if

$$\eqalign{{\mathrm{q \!\! -\!\!  ROFWA}}({{\mathcal N}^{\beth}}_1,{{\mathcal N}^{\beth}}_2, \ldots {{\mathcal N}^{\beth}}_n) &  = \sum\limits_{k = 1}^n {{\hbar ^\gamma }_k} {{\mathcal N}^{\beth}}_k \\  & = {\hbar ^\gamma }_1{{\mathcal N}^{\beth}}_1 \oplus {\hbar ^\gamma }_2{{\mathcal N}^{\beth}}_2 \oplus \ldots ,{\hbar ^\gamma }_n{{\mathcal N}^{\beth}}_n}$$

${\Lambda ^n}$ is a agglomeration of all q-ROFNs, and ${\hbar ^\gamma } = {({\hbar ^\gamma }_1,{\hbar ^\gamma }_2, \ldots ,{\hbar ^\gamma }_n)^T}$ is weight vector (WV) of $({{\mathcal N}^{\beth}}_1,{{\mathcal N}^{\beth}}_2, \ldots ,{{\mathcal N}^{\beth}}_\it n)$, with $0\le{\hbar ^\gamma }_k\le$ and $\sum\nolimits_{k = 1}^n {{\hbar ^\gamma }_k} = 1$.

**Theorem 3.6** Liu & Wang (2018): Let ${{\mathcal N}^{\beth}}_k = \langle {\mu _k},{\nu _k}\rangle$ be a q-ROFNs agglomeration and ${\mathrm{q \!\! -\!\!  ROFWA}}$ operator can also be determined as,

$$\mathrm{q-ROFWA}({{\mathcal N}^{{\mathrm{\beth}}}}_{\mathrm{1}},{{\mathcal N}^{{\mathrm{\beth}}}}_{\mathrm{2}}, \ldots {{\mathcal N}^{{\mathrm{\beth}}}}_{\mathrm{n}}){\mathrm{ = }}\left(\sqrt[{\mathrm{q}}]{\left({\mathrm{1 -}}\mathop{\widetilde{\prod}}_{{{k = 1}}}^{\mathrm{n}}{{({\mathrm{1 - }}\mu _{{k}}^{\mathrm{q}})}^{{\hbar ^\gamma }_{{k}}}}\right)},\;\mathop{\widetilde{\prod}}_{{\mathrm{k = 1}}}^{\rm n}\nu_{\mathrm{k}}^{{\hbar ^\gamma }_{\mathrm{k}}}\right) $$

**Definition 3.7** Liu & Wang (2018): Suppose that ${{\mathcal N}^{\beth}}_k = \langle {\mu _k},{\nu _k}\rangle$ be a agglomeration of q-ROFNs, and q-ROFWG: ${\Lambda ^n} \to \Lambda$, if

$$\eqalign{{\mathrm{q \!\! -\!\!  ROFWG}}({{\mathcal N}^{\beth}}_1,{{\mathcal N}^{\beth}}_2, \ldots {{\mathcal N}^{\beth}}_n)  & = \sum\limits_{k = 1}^n {{{\mathcal N}^{\beth}}_k^{{\hbar ^\gamma }_k}} \\  &  = {{\mathcal N}^{\beth}}_1^{{\hbar ^\gamma }_1} \otimes {{\mathcal N}^{\beth}}_2^{{\hbar ^\gamma }_2} \otimes \ldots ,{{\mathcal N}^{\beth}}_n^{{\hbar ^\gamma }_n}}$$

${\hbar ^\gamma } = {({\hbar ^\gamma }_1,{\hbar ^\gamma }_2, \ldots ,{\hbar ^\gamma }_n)^T}$ is a WV of $({{\mathcal N}^{\beth}}_1,{{\mathcal N}^{\beth}}_2, \ldots ,{{\mathcal N}^{\beth}}_\it n)$, such that $0\,{\le}\,{\hbar ^\gamma }_k\,{\le}\,1$, $\sum\nolimits_{k = 1}^n {{\hbar ^\gamma }_k} = 1$ and ${\Lambda ^n}$ be a agglomeration of all ${\mathrm{q \!\! -\!\!  ROFNs}}$.

**Theorem 3.8** Liu & Wang (2018): Let ${{\mathcal N}^{\beth}}_k = \langle {\mu _k},{\nu _k}\rangle$ is a agglomeration of q-ROFNs and q-ROFWG operator can be determined as,

$${\mathrm{q \!\! -\!\!  ROFWG}}({{\mathcal N}^{\beth}}_1,{{\mathcal N}^{\beth}}_2, \ldots {{\mathcal N}^{\beth}}_n) = \left( {\mathop {\tilde \prod }\limits_{{\mathrm{k = 1}}}^{\mathrm{n}} \mu _{\mathrm{k}}^{{\hbar ^\gamma }_{\mathrm{k}}},\root q \of {\left( {{\mathrm{1 - }}\tilde \prod _{{\mathrm{k = 1}}}^{\mathrm{n}}{{({\mathrm{1 - }}\nu _k^{\mathrm{q}})}^{{\hbar ^\gamma }_k}}} \right)} } \right)$$

### q-Rung orthopair fuzzy soft set

**Definition 3.9** Hamid, Riaz & Afzal (2020): Let *U* be a finite set of elements, *E* be a agglomeration of parameters, $H \subseteq E$ and $q \!\! -\!\!  RO{F^U}$ demonstrates the agglomeration of all subsets of q-ROFSS over *U*. A q-ROFSS is represented as $(\Omega ,H)$ or ${\Omega _H}$, where $\Omega :H \to q$- $RO{F^U}$ is a function, given as

$$\eqalign{{\Omega _H}  & = \{ ({ {\eth}},\{ \widetilde {\daleth},{\mu _{{\Omega _H}}}(\widetilde {\daleth}),{\nu _{{\Omega _H}}}(\widetilde {\daleth})\} ):{ {\eth}} \in H,\widetilde {\daleth} \in U\} \\  & = \left\{ \left({ {\eth}},\left\{ {{\widetilde {\daleth}} \over {({\mu _{{\Omega _H}}}(\widetilde {\daleth}),{\nu _{{\Omega _H}}}(\widetilde {\daleth}))}}\right\} \right):{ {\eth}} \in H,\widetilde {\daleth} \in U\right\} \\  &  = \left\{ \left({ {\eth}},\left\{ {{({\mu _{{\Omega _H}}}(\widetilde {\daleth}),{\nu _{{\Omega _H}}}(\widetilde {\daleth}))} \over {\widetilde {\daleth}}}\right\} \right):{ {\eth}} \in H,\widetilde {\daleth} \in U\right\}}$$

where ${\mu _{{\Omega _H}}}:U \to [0,1]$, ${\nu _{{\Omega _H}}}:U \to [0,1]$ be two functions, including the feature

$$0 \le \mu _{{\Omega _H}}^{\it q}(\widetilde {\daleth}) + \nu _{{\Omega _H}}^{\it q}(\widetilde {\daleth}) \le 1\qquad (q \ge 1)$$

Here, ${\mu _{{\Omega _H}}}(\widetilde {\daleth})$ and ${\nu _{{\Omega _H}}}(\widetilde {\daleth})$ demonstrates the PMV and NMV of the element $\widetilde {\daleth} \in U$. If ${\ddot \varpi_{i{\intercal}}} = {\mu _{{\Omega _H}}}({ {\eth}}_{\intercal})({\widetilde {\daleth}_i})$ and ${\widehat {\eth}_{i{\intercal}}} = {\nu _{{\Omega _H}}}({ {\eth}}_{\intercal})({\widetilde {\daleth}_i})$, then q-ROFSS ${\Omega _H}$ can be seen in Table 2.

**Table:** 

${ {\eth}}_{H}$	${\widetilde {\daleth}_1}$	${\widetilde {\daleth}_2}$	$\cdots$	${\widetilde {\daleth}_n}$
${ {\eth}}_{1}$	$\left\langle {{{\ddot \varpi }_{11}},{{\hat \Omega }_{11}}} \right\rangle $	$\left\langle {{{\ddot \varpi }_{12}},{{\hat \Omega }_{12}}} \right\rangle $	$\cdots$	$\left\langle {{{\ddot \varpi }_{1n}},{{\hat \Omega }_{1n}}} \right\rangle$
${ {\eth}}_{2}$	$\left\langle {{{\ddot \varpi }_{21}},{{\hat \Omega }_{21}}} \right\rangle$	$\left\langle {{{\ddot \varpi }_{22}},{{\hat \Omega }_{22}}} \right\rangle$	$\cdots$	$\left\langle {{{\ddot \varpi }_{2n}},{{\hat \Omega }_{2n}}} \right\rangle$
$\vdots$	$\vdots$	$\vdots$	$\ddots$	$\vdots$
${ {\eth}}_{m}$	$\left\langle {{{\ddot \varpi }_{m1}},{{\hat \Omega }_{m1}}} \right\rangle$	$\left\langle {{{\ddot \varpi }_{m2}},{{\hat \Omega }_{m2}}} \right\rangle$	$\cdots$	$\left\langle {{{\ddot \varpi }_{mn}},{{\hat \Omega }_{mn}}} \right\rangle$

and in matrix form as

$$\eqalign{
  & {\Omega _H} = {[({{\ddot \varpi }_{i{\intercal}}},{{\hat \Omega }_{i{\intercal}}})]_{m \times n}}\quad \;\;  \cr 
  & \,\,\,\,\,\,\, = \,\,\,\left( {\matrix{
   {\left\langle {{{\ddot \varpi }_{11}},{{\hat \Omega }_{11}}} \right\rangle } & {\left\langle {{{\ddot \varpi }_{12}},{{\hat \Omega }_{12}}} \right\rangle } &  \cdots  & {\left\langle {{{\ddot \varpi }_{1n}},{{\hat \Omega }_{1n}}} \right\rangle }  \cr 
   {\left\langle {{{\ddot \varpi }_{21}},{{\hat \Omega }_{21}}} \right\rangle } & {\left\langle {{{\ddot \varpi }_{22}},{{\hat \Omega }_{22}}} \right\rangle } &  \cdots  & {\left\langle {{{\ddot \varpi }_{2n}},{{\hat \Omega }_{2n}}} \right\rangle }  \cr 
    \vdots  &  \vdots  &  \ddots  &  \vdots   \cr 
   {\left\langle {{{\ddot \varpi }_{m1}},{{\hat \Omega }_{m1}}} \right\rangle } & {\left\langle {{{\ddot \varpi }_{m2}},{{\hat \Omega }_{m2}}} \right\rangle } &  \cdots  & {\left\langle {{{\ddot \varpi }_{mn}},{{\hat \Omega }_{mn}}} \right\rangle }  \cr 

 } } \right) \cr} $$

**Table 2:** 3-ROFSS $({\Omega _{\boldsymbol H}})$.

${\Omega _H}$	${\widetilde {\daleth}_1}$	${\widetilde {\daleth}_2}$	${\widetilde {\daleth}_3}$	${\widetilde {\daleth}_4}$	${\widetilde {\daleth}_5}$	${\widetilde {\daleth}_6}$
${T_1}$	$(0.80,0.40)$	$(0.70,0.30)$	$(0.50,0.20)$	$(0.90,0.60)$	$(0.90,0.50)$	$(0.90,0.50)$
${T_2}$	$(0.70,0.10)$	$(0.40,0.10)$	$(0.60,0.50)$	$(0.70,0.40)$	$(0.50,0.50)$	$(0.90,0.50)$
${T_3}$	$(0.80,0.10)$	$(0.60,0.20)$	$(0.40,0.30)$	$(0.80,0.50)$	$(0.90,0.70)$	$(0.90,0.50)$
${T_4}$	$(0.60,0.30)$	$(0.70,0.50)$	$(0.60,0.40)$	$(0.60,0.30)$	$(0.80,0.20)$	$(0.90,0.50)$
${T_5}$	$(0.70,0.50)$	$(0.70,0.30)$	$(0.60,0.30)$	$(0.60,0.40)$	$(0.70,0.90)$	$(0.90,0.50)$

**Definition 3.10** Hussain et al. (2020): Let ${{\mathcal N}^{\beth}}_{{ {\eth}}_{1j}} = \langle {\mu _{1j}},{\nu _{1j}}\rangle (\intercal = 1,2)$ and ${{\mathcal N}^{\beth}} = \langle \mu ,\nu \rangle$ be any three q-ROFSNs. Then

(1) $ {{{\bar{\mathcal N}}^{\beth}}} = \langle \nu ,\mu \rangle$

(2) ${{\mathcal N}^{\beth}}_{{ {\eth}}_{11}} \cup {{\mathcal N}^{\beth}}_{{ {\eth}}_{12}} = \langle {\mathrm{max}}\{ {\mu _{11}},{\mu _{12}}\} ,{\mathrm{min}}\{ {\nu _{11}},{\nu _{12}}\} \rangle$

(3) ${{\mathcal N}^{\beth}}_{{ {\eth}}_{11}} \cap {{\mathcal N}^{\beth}}_{{ {\eth}}_{12}} = \langle {\mathrm{min}}\{ {\mu _{11}},{\mu _{12}}\} ,{\mathrm{max}}\{ {\nu _{11}},{\nu _{12}}\} \rangle$

(4) ${{\mathcal N}^{\beth}}_{{ {\eth}}_{11}} \oplus {{\mathcal N}^{\beth}}_{{ {\eth}}_{12}} = \langle {(\mu _{{ {\eth}}_{11}}^{\it q} + \mu _{{ {\eth}}_{12}}^{\it q} - \mu _{{ {\eth}}_{11}}^{\it q}\mu _{12}^{\it q})^{1/q}},{\nu _{11}}{\nu _{12}}\rangle$

(5) ${{\mathcal N}^{\beth}}_{{ {\eth}}_{11}} \otimes {{\mathcal N}^{\beth}}_{{ {\eth}}_{12}} = \langle {\mu _{{ {\eth}}_{11}}}{\mu _{{ {\eth}}_{12}}},{(\nu _{{ {\eth}}_{11}}^{\it q} + \nu _{{ {\eth}}_{12}}^{\it q} - \nu _{{ {\eth}}_{11}}^{\it q}\nu _{{ {\eth}}_{12}}^{\it q})^{1/q}}\rangle$

(6) $\sigma {{\mathcal N}^{\beth}} = \langle {(1 - {(1 - {\mu ^{\it q}})^\sigma })^{1/q}},{\nu ^\sigma }\rangle$

(7) ${{\mathcal N}^{\beth}}^\sigma = \langle \mu _1^\sigma ,{(1 - {(1 - \nu _1^{\it q})^\sigma })^{1/q}}\rangle$

**Example 3.11** Consider ¥ $\, = \{ {\widetilde {\daleth}_1},{\widetilde {\daleth}_2},{\widetilde {\daleth}_3},{\widetilde {\daleth}_4},{\widetilde {\daleth}_5},{\widetilde {\daleth}_6}\}$ be the set of hostels and $\Lambda = \{ {T_1},{T_2},{T_3},{T_4},{T_5}\}$ be the set of attributes where

${T_1}$ parameter is for affordable,

${T_2}$ parameter is for clean,

${T_3}$ parameter is for good food,

${T_4}$ parameter is for capacious.

${T_5}$ parameter is for good location.

On the premise of the aforementioned criterion a decision expert weighed the options and documented their results in the form of q-ROFSNs as given in Table 2.

## Q-rofs prioritized weighted aggregation operators

The q-ROFS prioritized weighted averaging (q-ROFSPWA) operator and q-ROFS prioritized weighted geometric (q-ROFSPWG) operator are introduced in this section. The efficacious characteristics of the prospective operators are then given.

### q-ROFSPWA operator

**Definition 4.1** Assume that ${{\mathcal N}^{\beth}}_{{ {\eth}}_{i{\intercal}}} = \langle {\mu _{i{\intercal}}},{\nu _{i{\intercal}}}\rangle$, where $(\intercal = 1,2,..., {\it m\;{\mathrm{and}}\;i} = 1,2,...,\it n)$ is an agglomeration of q-ROFSNs, ${\hbar ^\gamma } = \{ {\hbar ^\gamma }_1,{\hbar ^\gamma }_2, \cdots {\hbar ^\gamma }_n\}$ and ${{{{\aleph}^{\it\ell}}_{\intercal}} \over {\sum\nolimits_{\intercal = 1}^n {{{\aleph}^{\it\ell}}_{\intercal}} }} = \boldsymbol\{ {{{{\aleph}^{\it\ell}}_1} \over {\sum\nolimits_{\intercal = 1}^n {{{\aleph}^{\it\ell}}_{\intercal}} }},{{{{\aleph}^{\it\ell}}_2} \over {\sum\nolimits_{\intercal = 1}^n {{{\aleph}^{\it\ell}}_{\intercal}} }}, \cdots ,{{{{\aleph}^{\it\ell}}_n} \over {\sum\nolimits_{\intercal = 1}^n {{{\aleph}^{\it\ell}}_{\intercal}} }}\boldsymbol\}$ are WVs for the parameters ${ {\eth}}_{\intercal}^{\prime}$ and decision makers ${{\mathcal D}_i}$ respectively with the conditions that $\sum\nolimits_{i = 1}^n {{\hbar ^\gamma }_i} = 1$ and $\sum\nolimits_{\intercal = 1}^m {{{{{\aleph}^{\it\ell}}_{\intercal}} \over {\sum\nolimits_{\intercal = 1}^n {{{\aleph}^{\it\ell}}_{\intercal}} }}} = 1$. Then the mapping for $q \!\! -\!\!  ROFSPWA:{\Omega ^n} \to \Omega$, be n-dimension mapping.

(1) $q \!\! -\!\!  ROFSPWA\left( {{{\mathcal N}^{\beth}}_{{ {\eth}}_{11}}, \ldots {{\mathcal N}^{\beth}}_{{ {\eth}}_{nm}}} \right) = \oplus _{k = 1}^m{\hbar ^\gamma }_k\left( {{{{{\aleph}^{\it\ell}}_1} \over {\sum\nolimits_{\intercal = 1}^n {{{\aleph}^{\it\ell}}_{\intercal}} }}{{\mathcal N}^{\beth}}_{{ {\eth}}_{11}} \oplus {{{{\aleph}^{\it\ell}}_2} \over {\sum\nolimits_{\intercal = 1}^n {{{\aleph}^{\it\ell}}_{\intercal}} }}{{\mathcal N}^{\beth}}_{{ {\eth}}_{12}} \oplus \ldots , \oplus {{{{\aleph}^{\it\ell}}_n} \over {\sum\nolimits_{\intercal = 1}^n {{{\aleph}^{\it\ell}}_{\intercal}} }}{{\mathcal N}^{\beth}}_{{ {\eth}}_{nm}}} \right)$

The q-ROFSPWA operator can also be considered by the theorem, as given below.

**Theorem 4.2** Assume that ${{\mathcal N}^{\beth}}_{i{\intercal}} = \langle {\mu _{i{\intercal}}},{\nu _{i{\intercal}}}\rangle$ be a agglomeration of q-ROFSNs, we can find $q \!\! -\!\!  ROFSPWA$ operator by,

(2) $$q \!\! -\!\!  ROFSPWA\left( {{{\mathcal N}^{\beth}}_{{ {\eth}}_{11}}, \ldots {{\mathcal N}^{\beth}}_{{ {\eth}}_{nm}}} \right) = \left( {\root q \of {1 - \widetilde \prod _{\intercal = 1}^m{{\left(\widetilde \prod _{i = 1}^n{{(1 - \mu _{i{\intercal}}^{\it q})}^{{{{{\aleph}^{\it\ell}}_{\intercal}} \over {\sum\nolimits_{\intercal = 1}^n {{{\aleph}^{\it\ell}}_{\intercal}} }}}}\right)}^{{\hbar ^\gamma }_{\intercal}}}} ,\mathop {\tilde \prod }\limits_{\intercal = 1}^n{{\left(\mathop {\tilde \prod }\limits_{\intercal = 1}^n\nu _{i{\intercal}}^{{{{{\aleph}^{\it\ell}}_{\intercal}} \over {\sum\nolimits_{\intercal = 1}^n {{{\aleph}^{\it\ell}}_{\intercal}} }}}\right)}^{{\hbar ^\gamma }_{\intercal}}}} \right)$$

As we know through operation laws, mathematical induction can be used to establish a specific result, that ${{\mathcal N}^{\beth}}_{{ {\eth}}_{11}} \oplus {{\mathcal N}^{\beth}}_{{{\eth}}_{12}} = \left( {\root q \of {{{({\mu _{11}})}^{\it q}} + {{({\mu _{12}})}^{\it q}} - {{({\mu _{11}})}^{\it q}}{{({\mu _{12}})}^{\it q}}} ,\;{\nu _{11}}{\nu _{12}}} \right)$ and $\lambda {{\mathcal N}^{\beth}} = (\root q \of {1 - [1 - {\mu ^{\it q}}]} ,{\nu ^\lambda })$ for $\lambda \ge 1$ We’ll start by showing that Eq. (1) is satisfied for n = 2 and m = 2, so we have

$$ {\eqalign  { & q - ROFSPWA\left( {{{\mathcal N}^{\beth}}_{\eth_{{11}}},{{\mathcal N}^{\beth}}_{\eth_{{12}}}} 
\right) 
\cr & =  \oplus _{\intercal = 1}^2{\hbar ^\gamma }_{\intercal}\left( { \oplus _{i = 1}^2{{{{\aleph}^{\ell}}_{\intercal}} \over {\sum\nolimits_{\intercal = 1}^n {{{\aleph}^{\ell}}_{\intercal}} }}{{\mathcal N}^{\beth}}_{\eth_{{i\intercal}}}} 
\right) \cr &
   = {\hbar ^\gamma }_1\left( { \oplus _{i = 1}^2{{{{\aleph}^{\ell}}_i} \over {\sum\nolimits_{i = 1}^n {{{\aleph}^{\ell}}_i} }}{{\mathcal N}^{\beth}}_{\eth_{{i1}}}} 
\right) \oplus {\hbar ^\gamma }_2\left( { \oplus _{i = 1}^2{{{{\aleph}^{\ell}}_i} \over {\sum\nolimits_{i = 1}^n {{{\aleph}^{\ell}}_i} }}{{\mathcal N}^{\beth}}_{\eth_{{i2}}}} 
\right)
\cr &  = {\hbar ^\gamma }_1\left( {{{{{\aleph}^{\ell}}_1} \over {\sum\nolimits_{i = 1}^n {{{\aleph}^{\ell}}_i} }}{{\mathcal N}^{\beth}}_{\eth_{{11}}} \oplus {{{{\aleph}^{\ell}}_2} \over {\sum\nolimits_{i = 1}^n {{{\aleph}^{\ell}}_i} }}{{\mathcal N}^{\beth}}_{\eth_{{21}}}} 
\right) \oplus {\hbar ^\gamma }_2\left( {{{{{\aleph}^{\ell}}_2} \over {\sum\nolimits_{i = 1}^n {{{\aleph}^{\ell}}_i} }}{{\mathcal N}^{\beth}}_{\eth_{{12}}} \oplus {{{{\aleph}^{\ell}}_2} \over {\sum\nolimits_{i = 1}^n {{{\aleph}^{\ell}}_i} }}{{\mathcal N}^{\beth}}_{\eth_{{22}}}} 
\right) \cr &
   = {\hbar ^\gamma }_1\left\{ {\left( {
\root q \of {1 - {{(1 - \mu _{11}^q)}^{{{{{\aleph}^{\ell}}_{\intercal}} \over {\sum\nolimits_{\intercal = 1}^n {{{\aleph}^{\ell}}_{\intercal}} }}}}} ,
u _{11}^{{{{{\aleph}^{\ell}}_{\intercal}} \over {\sum\nolimits_{\intercal = 1}^n {{{\aleph}^{\ell}}_{\intercal}} }}}} 
\right) \oplus \left( {
\root q \of {1 - {{(1 - \mu _{21}^q)}^{{{{{\aleph}^{\ell}}_{\intercal}} \over {\sum\nolimits_{\intercal = 1}^n {{{\aleph}^{\ell}}_{\intercal}} }}}}} ,
u _{21}^{{{{{\aleph}^{\ell}}_{\intercal}} \over {\sum\nolimits_{\intercal = 1}^n {{{\aleph}^{\ell}}_{\intercal}} }}}} 
\right)} 
\right\} 
\cr &  \oplus {\hbar ^\gamma }_2\left\{ {\left( {
\root q \of {1 - {{(1 - \mu _{12}^q)}^{{{{{\aleph}^{\ell}}_{\intercal}} \over {\sum\nolimits_{\intercal = 1}^n {{{\aleph}^{\ell}}_{\intercal}} }}}}} ,
u _{12}^{{{{{\aleph}^{\ell}}_{\intercal}} \over {\sum\nolimits_{\intercal = 1}^n {{{\aleph}^{\ell}}_{\intercal}} }}}} 
\right) \oplus \left( {
\root q \of {1 - {{(1 - \mu _{22}^q)}^{{{{{\aleph}^{\ell}}_{\intercal}} \over {\sum\nolimits_{\intercal = 1}^n {{{\aleph}^{\ell}}_{\intercal}} }}}}} ,
u _{22}^{{{{{\aleph}^{\ell}}_{\intercal}} \over {\sum\nolimits_{\intercal = 1}^n {{{\aleph}^{\ell}}_{\intercal}} }}}} 
\right)} 
\right\} 
\cr &  = {\hbar ^\gamma }_1\left( {
\root q \of {1 - \widetilde \prod _{i = 1}^2{{(1 - \mu _{i1}^q)}^{{{{{\aleph}^{\ell}}_i} \over {\sum\nolimits_{i = 1}^n {{{\aleph}^{\ell}}_i} }}}}} ,\mathop{\widetilde \prod} _{i = 1}^2
u _{i1}^{{{{{\aleph}^{\ell}}_i} \over {\sum\nolimits_{i = 1}^2 {{{\aleph}^{\ell}}_i} }}}} 
\right) 
   \oplus {\hbar ^\gamma }_2\left( {
\root q \of {1 - \widetilde \prod _{i = 1}^2{{(1 - \mu _{i2}^q)}^{{{{{\aleph}^{\ell}}_i} \over {\sum\nolimits_{i = 1}^n {{{\aleph}^{\ell}}_i} }}}}} ,\mathop{\widetilde \prod} _{i = 1}^2
u _{i2}^{{{{{\aleph}^{\ell}}_i} \over {\sum\nolimits_{i = 1}^2 {{{\aleph}^{\ell}}_i} }}}} 
\right) \cr &
   = {(
\root q \of {1 - (\widetilde \prod _{i = 1}^2{{(1 - \mu _{i1}^q)}^{{{{{\aleph}^{\ell}}_i} \over {\sum\nolimits_{i = 1}^n {{{\aleph}^{\ell}}_i} }}}}} )^{{\hbar ^\gamma }_1}},{(
u _{i1}^{{{{{\aleph}^{\ell}}_i} \over {\sum\nolimits_{i = 1}^2 {{{\aleph}^{\ell}}_i} }}})^{{\hbar ^\gamma }_1}}) 
   \oplus {(
\root q \of {1 - (\widetilde \prod _{i = 1}^2{{(1 - \mu _{i2}^q)}^{{{{{\aleph}^{\ell}}_i} \over {\sum\nolimits_{i = 1}^n {{{\aleph}^{\ell}}_i} }}}}} )^{{\hbar ^\gamma }_2}},{(
u _{i2}^{{{{{\aleph}^{\ell}}_i} \over {\sum\nolimits_{i = 1}^2 {{{\aleph}^{\ell}}_i} }}})^{{\hbar ^\gamma }_2}}) \cr &
   = {(
\root q \of {1 - \widetilde \prod _{\intercal = 1}^2(\widetilde \prod _{i = 1}^2{{(1 - \mu _{i\intercal}^q)}^{{{{{\aleph}^{\ell}}_i} \over {\sum\nolimits_{i = 1}^n {{{\aleph}^{\ell}}_i} }}}}} )^{{\hbar ^\gamma }_{\intercal}}},\mathop{\widetilde \prod} _{\intercal = 1}^2{(\mathop{\widetilde \prod} _{i = 1}^2
u _{i\intercal}^{{{{{\aleph}^{\ell}}_i} \over {\sum\nolimits_{i = 1}^2
{{{\aleph}^{\ell}}_i} }}})^{{\hbar ^\gamma }_{\intercal}}})  }}$$

As a result, the conclusion holds for n = 2, m = 2. Assume that Eq. (1) is true for $n = {k_1}$, $m = {k_2}$

$$ q \!\! -\!\!  ROFSPWA\left( {{{\mathcal N}^{\beth}}_{{ {\eth}}_{11}}, \ldots {{\mathcal N}^{\beth}}_{{ {\eth}}{_{{k_1}{k_2}}}}} \right) = \left( {\root q \of {1 - \widetilde \prod _{\intercal = 1}^{{k_2}}{{\left( {\widetilde \prod _{i = 1}^{{k_1}}{{(1 - \mu _{i{\intercal}}^{\it q})}^{{{{{\aleph}^{\it\ell}}_i} \over {\sum\nolimits_{i = 1}^n {{{\aleph}^{\it\ell}}_i} }}}}} \right)}^{{\hbar ^\gamma }_{\intercal}}}} ,\mathop {\mathop \prod \limits^ \sim  }\limits_{\intercal = 1}^{{k_2}}{{\boldsymbol(\mathop {\mathop \prod \limits^ \sim  }\limits_{i = 1}^{{k_1}}\nu _{i{\intercal}}^{{{{{\aleph}^{\it\ell}}_i} \over {\sum\nolimits_{i = 1}^n {{{\aleph}^{\it\ell}}_i} }}}\boldsymbol)}^{{\hbar ^\gamma }_{\intercal}}}} \right)$$

Assume that Eq. (1) holds for $n = {k_1} + 1$, $m = {k_2} + 1.$

$$\eqalign{ q \!\! -\!\!  ROFSPWA({{\mathcal N}^{\beth}}_{{ {\eth}}_{11}}, &   \ldots {{\mathcal N}^{\beth}}_{{ {\eth}}{_{({k_1} + 1)({k_2} + 1)}}}) \\  & = \left\{ { \oplus _{\intercal = 1}^{{k_2}}{\hbar ^\gamma }_{\intercal}\left( { \oplus _{i = 1}^{{k_1}}{{{{\aleph}^{\it\ell}}_i} \over {\sum\nolimits_{i = 1}^n {{{\aleph}^{\it\ell}}_i} }}{{\mathcal N}^{\beth}}_{{ {\eth}}_{i{\intercal}}}} \right)} \right\} \oplus {\hbar ^\gamma }_{({k_1} + 1)}\left( {{{{{{{\aleph}^{\it\ell}}_i} \over {\sum\nolimits_{i = 1}^n {{{\aleph}^{\it\ell}}_i} }}}_{({k_2} + 1)}}{{\mathcal N}^{\beth}}_{{ {\eth}}{_{({k_1} + 1)({k_2} + 1)}}}} \right)\\   &  =  \left( {\root q \of {1 - \widetilde \prod _{\intercal = 1}^{{k_2}}{{\left(\widetilde \prod _{i = 1}^{{k_1}}{{(1 - \mu _{i{\intercal}}^{\it q})}^{{{{{\aleph}^{\it\ell}}_i} \over {\sum\nolimits_{i = 1}^n {{{\aleph}^{\it\ell}}_i} }}}}\right)}^{{\hbar ^\gamma }_{\intercal}}}} ,\mathop {\mathop \prod \limits^ \sim  }\limits_{\intercal = 1}^{{k_2}}{{\left(\mathop {\mathop \prod \limits^ \sim  }\limits_{i = 1}^{{k_1}}\nu _{i{\intercal}}^{{{{{\aleph}^{\it\ell}}_i} \over {\sum\nolimits_{i = 1}^n {{{\aleph}^{\it\ell}}_i} }}}\right)}^{{\hbar ^\gamma }_{\intercal}}}} \right)\\  &  \oplus {\hbar ^\gamma }_{({k_1} + 1)}\left( {{{{{{{\aleph}^{\it\ell}}_i} \over {\sum\nolimits_{i = 1}^n {{{\aleph}^{\it\ell}}_i} }}}_{({k_2} + 1)}}{{\mathcal N}^{\beth}}_{{ {\eth}}{_{({k_1} + 1)({k_2} + 1)}}}} \right)\\  &  = \left( {\root q \of {1 - \widetilde \prod _{\intercal = 1}^{({k_2} + 1)}{{\left(\widetilde \prod _{i = 1}^{({k_1} + 1)}{{(1 - \mu _{i{\intercal}}^{\it q})}^{{{{{\aleph}^{\it\ell}}_i} \over {\sum\nolimits_{i = 1}^n {{{\aleph}^{\it\ell}}_i} }}}}\right)}^{{\hbar ^\gamma }_{\intercal}}}} ,\;\mathop {\mathop \prod \limits^ \sim  }\limits_{\intercal = 1}^{{k_2} + 1} {{\left(\;\mathop {\mathop \prod \limits^ \sim  }\limits_{i = 1}^{{k_1} + 1}\nu _{i{\intercal}}^{{{{{\aleph}^{\it\ell}}_i} \over {\sum\nolimits_{i = 1}^n {{{\aleph}^{\it\ell}}_i} }}}\right)}^{{\hbar ^\gamma }_{\intercal}}}} \right)}$$

Thus, Eq. (1) holds for $n = {k_1} + 1$, $m = {k_2} + 1$. As a result, Eq. (1) holds for every $m,n \ge 1,$ through mathematical induction. Furthermore, to demonstrate that the aggregated result of q-ROFSPWA is also a q-ROPFSN. Any ${{\mathcal N}^{\beth}}_{{ {\eth}}_{i{\intercal}}} =$ $({\mu _{i{\intercal}}},{\nu _{i{\intercal}}}),(i = 1,2,...,n\;{\mathrm{and}}\;\intercal = 1,2,...,\it n)$, where $0 \le {\mu _{i{\intercal}}},\;{\nu _{i{\intercal}}} \le 1,$ satisfying that $0 \le \mu _{i{\intercal}}^{\it q} + \nu _{i{\intercal}}^{\it q} \le 1$ with WVs ${\hbar ^\gamma } = \{ {\hbar ^\gamma }_1,{\hbar ^\gamma }_2, \cdots {\hbar ^\gamma }_n\}$ and ${{{{\aleph}^{\it\ell}}_{\intercal}} \over {\sum\nolimits_{\intercal = 1}^n {{{\aleph}^{\it\ell}}_{\intercal}} }} = \boldsymbol\{ {{{{{\aleph}^{\it\ell}}_1} \over {\sum\nolimits_{\intercal = 1}^n {{{\aleph}^{\it\ell}}_{\intercal}} }}_1},{{{{{\aleph}^{\it\ell}}_2} \over {\sum\nolimits_{\intercal = 1}^n {{{\aleph}^{\it\ell}}_{\intercal}} }}_2}, \cdots ,{{{{{\aleph}^{\it\ell}}_n} \over {\sum\nolimits_{\intercal = 1}^n {{{\aleph}^{\it\ell}}_{\intercal}} }}_m}\boldsymbol\}$for the parameters ${ {\eth}}_{\intercal}$ and decision makers ${{\mathcal D}_i}$ respectively with the conditions that $\sum\nolimits_{i = 1}^n {{{{{\aleph}^{\it\ell}}_i} \over {\sum\nolimits_{i = 1}^n {{{\aleph}^{\it\ell}}_i} }}} = 1$ and $\sum\nolimits_{\intercal = 1}^m {{\hbar ^\gamma }_{\intercal}} = 1$.

As

$$\eqalign{0 \le {\mu _{i{\intercal}}} \le 1  &  \Rightarrow 0 \le 1 - {\mu _{i{\intercal}}} \le 1 \Rightarrow 0 \le {\left( {1 - \mu _{i{\intercal}}^{\it q}} \right)^{{{{{\aleph}^{\it\ell}}_i} \over {\sum\nolimits_{i = 1}^n {{{\aleph}^{\it\ell}}_i} }}}} \le 1\\  &  \Rightarrow 0 \le \mathop {\tilde \prod }\limits_{i = 1}^n(1 - \mu _{i{\intercal}}^{\it q}) \le 1 \Rightarrow 0 \le \mathop {\tilde \prod }\limits_{\intercal = 1}^m{\left( {\mathop {\tilde \prod }\limits_{i = 1}^n{{(1 - \mu _{i{\intercal}}^{\it q})}^{{{{{\aleph}^{\it\ell}}_i} \over {\sum\nolimits_{i = 1}^n {{{\aleph}^{\it\ell}}_i} }}}}} \right)^{{\hbar ^\gamma }_{\intercal}}} \le 1\\  &  \Rightarrow 0 \le \sqrt {\widetilde \prod _{\intercal = 1}^m{{\left( {\widetilde \prod _{i = 1}^n{{(1 - \mu _{i{\intercal}}^{\it q})}^{{{{{\aleph}^{\it\ell}}_i} \over {\sum\nolimits_{i = 1}^n {{{\aleph}^{\it\ell}}_i} }}}}} \right)}^{{\hbar ^\gamma }_{\intercal}}}} \le 1}$$

Similarly,

$$\eqalign{0 \le {\mu
_{i{\intercal}}} \le 1  &  \Rightarrow 0 \le \mathop {\widetilde \prod} _{i = 1}^{ n} \nu _{i{\intercal}}^{{{{{\aleph}^{\it\ell}}_i} \over {\sum\nolimits_{i = 1}^n {{{\aleph}^{\it\ell}}_i} }}} \le 1 \\  & \Rightarrow 0 \le \mathop{\widetilde \prod} _{\intercal = 1}^{ m}{\left( {\mathop{\widetilde \prod} _{i = 1}^{ n}\nu _{i{\intercal}}^{{{{{\aleph}^{\it\ell}}_i} \over {\sum\nolimits_{i = 1}^n {{{\aleph}^{\it\ell}}_i} }}}} \right)^{{\hbar ^\gamma }_{\intercal}}} \le 1 \\   \mu _{i{\intercal}}^{\it q} + \nu _{i{\intercal}}^{\it q} \le 1 & \Rightarrow \nu _{i{\intercal}}^{\it q} \le 1 - \mu _{i{\intercal}}^{\it q}\\  &  \Rightarrow \mathop{\widetilde \prod} _{ i = 1}^{ n}{\left( {\nu _{i{\intercal}}^{\it q}} \right)^{{{{{\aleph}^{\it\ell}}_i} \over {\sum\nolimits_{i = 1}^n {{{\aleph}^{\it\ell}}_i} }}}} \le \left( {\mathop{\widetilde \prod} _{ i = 1}^{ n}{{(1 - \mu _{i{\intercal}}^{\it q})}^{{{{{\aleph}^{\it\ell}}_i} \over {\sum\nolimits_{i = 1}^n {{{\aleph}^{\it\ell}}_i} }}}}} \right)\\  &  \Rightarrow {\left( {\mathop{\widetilde \prod} _{ \intercal = 1}^{ m}{{\left(\mathop{\widetilde \prod} _{ i = 1}^{ n}\nu _{i{\intercal}}^{\it q}\right)}^{{{{{\aleph}^{\it\ell}}_i} \over {\sum\nolimits_{i = 1}^n {{{\aleph}^{\it\ell}}_i} }}}}} \right)^{{\hbar ^\gamma }_{\intercal}}} \le \mathop{\widetilde \prod} _{ \intercal = 1}^{ m}{\left( {\mathop{\widetilde \prod} _{ i = 1}^{ n}{{(1 - \mu _{i{\intercal}}^{\it q})}^{{{{{\aleph}^{\it\ell}}_i} \over {\sum\nolimits_{i = 1}^n {{{\aleph}^{\it\ell}}_i} }}}}} \right)^{{\hbar ^\gamma }_{\intercal}}}\\  &  \Rightarrow {\left( {\mathop{\widetilde \prod} _{ \intercal = 1}^{ m}{{\left(\mathop{\widetilde \prod} _{ i = 1}^{ n}\nu _{i{\intercal}}^{{{{{\aleph}^{\it\ell}}_i} \over {\sum\nolimits_{i = 1}^n {{{\aleph}^{\it\ell}}_i} }}}\right)}^{{\hbar ^\gamma }_{\intercal}}}} \right)^{\it q}} \le \mathop{\widetilde \prod} _{ \intercal = 1}^{ m}{\left( {\mathop{\widetilde \prod} _{ i = 1}^{ n}{{(1 - \mu _{i{\intercal}}^{\it q})}^{{{{{\aleph}^{\it\ell}}_i} \over {\sum\nolimits_{i = 1}^n {{{\aleph}^{\it\ell}}_i} }}}}} \right)^{{\hbar ^\gamma }_{\intercal}}}}$$

$$0 \le \root q \of {\boldsymbol\{ 1 - \widetilde \prod _{\intercal = 1}^m{{\left(\widetilde \prod _{i = 1}^n{{(1 - \mu _{i{\intercal}}^{\it q})}^{{{{{\aleph}^{\it\ell}}_i} \over {\sum\nolimits_{i = 1}^n {{{\aleph}^{\it\ell}}_i} }}}}\right)}^{{\hbar ^\gamma }_{\intercal}}}} {\boldsymbol\} ^{\it q}} + {\left\{ \mathop {\tilde \prod }\limits_{\intercal = 1}^m{\left(\mathop {\tilde \prod }\limits_{i = 1}^n\nu _{i{\intercal}}^{{{{{\aleph}^{\it\ell}}_i} \over {\sum\nolimits_{i = 1}^n {{{\aleph}^{\it\ell}}_i} }}}\right)^{{\hbar ^\gamma }_{\intercal}}}\right\} ^{\it q}}$$

By Eq. (2), we have

$$\le 1 - \mathop {\tilde \prod }\limits_{\intercal = 1}^m{\left(\mathop {\tilde \prod }\limits_{i = 1}^n{\left(1 - \mu _{i{\intercal}}^{\it q}\right)^{{{{{\aleph}^{\it\ell}}_i} \over {\sum\nolimits_{i = 1}^n {{{\aleph}^{\it\ell}}_i} }}}}\right)^{{\hbar ^\gamma }_{\intercal}}} + \mathop {\tilde \prod }\limits_{\intercal = 1}^m{\left(\mathop {\tilde \prod }\limits_{i = 1}^n{(1 - \mu _{i{\intercal}}^{\it q})^{{{{{\aleph}^{\it\ell}}_i} \over {\sum\nolimits_{i = 1}^n {{{\aleph}^{\it\ell}}_i} }}}}\right)^{{\hbar ^\gamma }_{\intercal}}} = 1$$

$$0 \le \root q \of {\boldsymbol\{ 1 - \widetilde \prod _{\intercal = 1}^m{{\left(\widetilde \prod _{i = 1}^n{{(1 - \mu _{i{\intercal}}^{\it q})}^{{{{{\aleph}^{\it\ell}}_i} \over {\sum\nolimits_{i = 1}^n {{\hbar ^\gamma }_i} }}}}\right)}^{{\hbar ^\gamma }_{\intercal}}}} {\boldsymbol\} ^{\it q}} + {\left\{ \mathop {\tilde \prod }\limits_{\intercal = 1}^m{\left(\mathop {\tilde \prod }\limits_{i = 1}^n\nu _{i{\intercal}}^{{{{{\aleph}^{\it\ell}}_i} \over {\sum\nolimits_{i = 1}^n {{{\aleph}^{\it\ell}}_i} }}}\right)^{{\hbar ^\gamma }_{\intercal}}}\right\} ^{\it q}} \le 1$$

As a result, the aggregated result obtained by the q-ROFSPWA operator is actually a q-ROFSN.

**Theorem 4.3** Consider a set of q-ROFSNs ${{\mathcal N}^{\beth}}_{{ {\eth}}_{i{\intercal}}} = \langle {\mu _{i{\intercal}}},{\nu _{i{\intercal}}}\rangle \;(i = 1,2,...,n\;{\mathrm{and}}\;\intercal$ $\intercal = 1,2,...\it \it m)$ with WVs ${{{{\aleph}^{\it\ell}}_{\intercal}} \over {\sum\nolimits_{\intercal = 1}^n {{{\aleph}^{\it\ell}}_{\intercal}} }} = {\left\{ {{{{{\aleph}^{\it\ell}}_1} \over {\sum\nolimits_{\intercal = 1}^n {{{\aleph}^{\it\ell}}_{\intercal}} }}_1},{{{{{\aleph}^{\it\ell}}_2} \over {\sum\nolimits_{\intercal = 1}^n {{{\aleph}^{\it\ell}}_{\intercal}} }}_2}, \cdots ,{{{{{\aleph}^{\it\ell}}_n} \over {\sum\nolimits_{\intercal = 1}^n {{{\aleph}^{\it\ell}}_{\intercal}} }}_n}\right\} ^T}$ and ${\hbar ^\gamma } = {\{ {\hbar ^\gamma }_1,{\hbar ^\gamma }_2, \cdots {\hbar ^\gamma }_m\} ^T}$ for the decision makers ${{\mathcal D}_i}$ and for the parameters ${ {\eth}}_{\intercal}^{\prime}$ respectively with the conditions that $\sum\nolimits_{i = 1}^n {{{{{{{\aleph}^{\it\ell}}_{\intercal}} \over {\sum\nolimits_{\intercal = 1}^n {{{\aleph}^{\it\ell}}_{\intercal}} }}}_i}} = 1$ and $\sum\nolimits_{\intercal = 1}^m {{\hbar ^\gamma }_{\intercal}} = 1.$ Then the q-ROFSPWA operator holds the following properties:

(**Idempotency**): If ${{\mathcal N}^{\beth}}_{{ {\eth}}_{i{\intercal}}} = {\Gamma _e}(\forall i = 1,2,...,n\;{\mathrm{and}}\;\intercal = 1,2,...,\it \it m)$, where ${\Gamma _e} = (p,r)$, then

$$q \!\! -\!\!  ROFSPWA\left( {{{\mathcal N}^{\beth}}_{11},{{\mathcal N}^{\beth}}_{12}, \ldots {{\mathcal N}^{\beth}}_{nm}} \right) = {\Gamma _e}$$

(**Boundedness**): If ${{\mathcal N}^{\beth}}_{{ {\eth}}_{i{\intercal}}}^ - = \left( {{\mathrm{mi}}{{\mathrm{n}}_{\intercal}}\;{\mathrm{mi}}{{\mathrm{n}}_i}\{ {\mu _{i{\intercal}}}\} ,\;{\mathrm{ma}}{{\mathrm{x}}_{\intercal}}\;{\mathrm{ma}}{{\mathrm{x}}_i}\{ {\nu _{i{\intercal}}}\} } \right)$ and ${{\mathcal N}^{\beth}}_{{ {\eth}}_{i{\intercal}}}^ + = ({\mathrm{ma}}{{\mathrm{x}}_{\intercal}}{\mathrm{ma}}{{\mathrm{x}}_i}\{ {\mu _{i{\intercal}}}\} ,\;{\mathrm{mi}}{{\mathrm{n}}_{\intercal}}\;{\mathrm{mi}}{{\mathrm{n}}_i}\{ {\nu _{i{\intercal}}}\} ){\rm  , then}$

$${{\mathcal N}^{\beth}}_{{{\eth}}_{i{\intercal}}}^ - \le q \!\! -\!\!  ROFSPWA\left( {{{\mathcal N}^{\beth}}_{{ {\eth}}_{11}},{{\mathcal N}^{\beth}}_{{ {\eth}}_{12}},{{\mathcal N}^{\beth}}_{{ {\eth}}_{13}},...,{{\mathcal N}^{\beth}}_{{ {\eth}}_{nm}}} \right) \le {{\mathcal N}^{\beth}}_{{ {\eth}}_{i{\intercal}}}^ +$$

(**Monotonicity**): If ${\Gamma _{{e_{i{\intercal}}}}} = ({p_{i{\intercal}}},{r_{i{\intercal}}})$, $(\forall i = 1,2,...,n\;{\mathrm{and}}\;\intercal = 1,2,...,\it \it m)$ be the agglomeration of q-ROFSNs such that ${\mu _{i{\intercal}}} \le {p_{i{\intercal}}}$ and ${\nu _{i{\intercal}}} \ge {r_{i{\intercal}}}$ then

$$q \!\! -\!\!  ROFSPWA\left( {{{\mathcal N}^{\beth}}_{11},{{\mathcal N}^{\beth}}_{12}, \ldots {{\mathcal N}^{\beth}}_{nm}} \right) \le q \!\! -\!\!  ROFSPWA\left( {{\Gamma _{11}},{\Gamma _{12}}, \ldots {\Gamma _{nm}}} \right)$$

(**Shift Invariance**): If ${\tilde \Gamma _e} = (p,r)$ is another q-ROFSN, then

$$ q \!\! -\!\!  ROFSPWA\left( {{{\mathcal N}^{\beth}}_{{ {\eth}}_{11}} \oplus {\Gamma _e},{{\mathcal N}^{\beth}}_{{ {\eth}}_{12}} \oplus {\Gamma _e},...{{\mathcal N}^{\beth}}_{{ {\eth}}_{nm}} \oplus {\Gamma _e}} \right) = q \!\! -\!\!  ROFSPWA\left( {{{\mathcal N}^{\beth}}_{{ {\eth}}_{11}},{{\mathcal N}^{\beth}}_{{ {\eth}}_{12}}, \ldots {{\mathcal N}^{\beth}}_{{ {\eth}}_{nm}}} \right) \oplus {\Gamma _e}$$

(**Homogeneity**): If $\lambda$ is any real number such that $\lambda \ge 0$, then

$$ q \!\! -\!\!  ROFSPWA\left( {\lambda {{\mathcal N}^{\beth}}_{{\widetilde {\eth}}_{11}},\lambda {{\mathcal N}^{\beth}}_{{ {\eth}}_{12}},...\lambda {{\mathcal N}^{\beth}}_{{ {\eth}}_{nm}}} \right) = \lambda \;q \!\! -\!\!  ROFSPWA\left( {{{\mathcal N}^{\beth}}_{{ {\eth}}_{11}},{{\mathcal N}^{\beth}}_{{ {\eth}}_{12}},...{{\mathcal N}^{\beth}}_{{ {\eth}}_{nm}}} \right)$$

(**Idempotency**): As it is given that if for all ${{\mathcal N}^{\beth}}_{{ {\eth}}_{i{\intercal}}} = {\Gamma _e} = (p,r)(\forall \;i = 1,2,...,n$ and $\intercal = 1,2,...\it \it m)$, then from Theorem 1, we have

$q \!\! -\!\!  ROFSPWA({{\mathcal N}^{\beth}}_{{ {\eth}}_{11}},{{\mathcal N}^{\beth}}_{{ {\eth}}_{12}}, \ldots {{\mathcal N}^{\beth}}_{{ {\eth}}_{nm}})$

$$\eqalign{ &  =  \left( {\root q \of {1 - \widetilde \prod _{\intercal = 1}^m{{\left(\widetilde \prod _{i = 1}^n{{(1 - \mu _{i{\intercal}}^{\it q})}^{{{{{\aleph}^{\it\ell}}_i} \over {\sum\nolimits_{i = 1}^n {{{\aleph}^{\it\ell}}_i} }}}}\right)}^{{\hbar ^\gamma }_{\intercal}}}} ,\mathop {\tilde \prod }\limits_{\intercal = 1}^m{{\left(\mathop {\tilde \prod }\limits_{i = 1}^n\nu _{i{\intercal}}^{{{{{\aleph}^{\it\ell}}_i} \over {\sum\nolimits_{i = 1}^n {{{\aleph}^{\it\ell}}_i} }}}\right)}^{{\hbar ^\gamma }_{\intercal}}}} \right)\\  &  = \left( {\root q \of {1 - \widetilde \prod _{\intercal = 1}^m{{\left(\widetilde \prod _{i = 1}^n{{(1 - p_{i{\intercal}}^{\it q})}^{{{{{\aleph}^{\it\ell}}_i} \over {\sum\nolimits_{i = 1}^n {{{\aleph}^{\it\ell}}_i} }}}}\right)}^{{\hbar ^\gamma }_{\intercal}}}} ,\mathop {\tilde \prod }\limits_{\intercal = 1}^m{{\left(\mathop {\tilde \prod }\limits_{i = 1}^nr_{i{\intercal}}^{{{{{\aleph}^{\it\ell}}_i} \over {\sum\nolimits_{i = 1}^n {{{\aleph}^{\it\ell}}_i} }}}\right)}^{{\hbar ^\gamma }_{\intercal}}}} \right)\\  &  = \left( {\root q \of {1 - (1 - {p^{\it q}})} ,r} \right) = (p,\;r) = \widetilde {{\Gamma _e}}}$$

Therefore,

$$q \!\! -\!\!  ROFSPWA\left( {{{\mathcal N}^{\beth}}_{{ {\eth}}_{11}},{{\mathcal N}^{\beth}}_{{ {\eth}}_{12}},\; \ldots {{\mathcal N}^{\beth}}_{{ {\eth}}_{nm}}} \right) = {\Gamma _e}$$

(**Boundedness**): As ${{\mathcal N}^{\beth}}_{{ {\eth}}_{i{\intercal}}}^ - = \left( {{\mathrm{mi}}{{\mathrm{n}}_{\intercal}}\;{\mathrm{mi}}{{\mathrm{n}}_i}\{ {\mu _{i{\intercal}}}\} ,\;{\mathrm{ma}}{{\mathrm{x}}_{\intercal}}\;{\mathrm{ma}}{{\mathrm{x}}_i}\{ {\nu _{i{\intercal}}}\} } \right)$ and ${{\mathcal N}^{\beth}}_{{ {\eth}}_{i{\intercal}}}^ + = ({\mathrm{ma}}{{\mathrm{x}}_{\intercal}}\;{\mathrm{ma}}{{\mathrm{x}}_i}\{ {\mu _{i{\intercal}}}\} ,\;{\mathrm{mi}}{{\mathrm{n}}_{\intercal}}\;{\mathrm{mi}}{{\mathrm{n}}_i}\{ {\nu _{i{\intercal}}}\} )$

To prove that

$${{\mathcal N}^{\beth}}_{{ {\eth}}_{i{\intercal}}}^ - \le q \!\! -\!\!  ROFSPWA\left( {{{\mathcal N}^{\beth}}_{{ {\eth}}_{11}},{{\mathcal N}^{\beth}}_{{ {\eth}}_{12}},{{\mathcal N}^{\beth}}_{{ {\eth}}_{13}},...,{{\mathcal N}^{\beth}}_{{ {\eth}}_{nm}}} \right) \le {{\mathcal N}^{\beth}}_{{ {\eth}}_{i{\intercal}}}^ +$$

Now for each $i = 1,2,...,n$ and $\intercal = 1,2,...,\it m$, we have

$$
\eqalign{ {\mathrm{mi}}{{\mathrm{n}}_{\intercal}}\;{\mathrm{mi}}{{\mathrm{n}}_i}\{
{\mu _{i{\intercal}}}\}  &  \le {\mu _{i{\intercal}}}\le
{\mathrm{ma}}{{\mathrm{x}}_{\intercal}}\;{\mathrm{ma}}{{\mathrm{x}}_i}\{ {\mu
_{i{\intercal}}}\} \cr  &  \Leftrightarrow 1 -
{\mathrm{ma}}{{\mathrm{x}}_{\intercal}}\;{\mathrm{ma}}{{\mathrm{x}}_i}\{ \mu _{i{\intercal}}^{\it q}\} \Leftrightarrow 1 - \mu _{i{\intercal}}^{\it q} \le {\mathrm{mi}}{{\mathrm{n}}_{\intercal}}\;{\mathrm{mi}}{{\mathrm{n}}_i}\{ \mu _{i{\intercal}}^{\it q}\} \cr  &  \Leftrightarrow \mathop {\tilde \prod}\limits _{ \intercal = 1}^{ m}{\left(\mathop {\tilde \prod}\limits _{ i = 1}^{ n}{(1 - {\mathrm{ma}}{{\mathrm{x}}_{\intercal}}\;{\mathrm{ma}}{{\mathrm{x}}_i}\{ \mu _{i{\intercal}}^{\it q}\} )^{{{{{\aleph}^{\it\ell}}_i} \over {\sum\nolimits_{i = 1}^n {{{\aleph}^{\it\ell}}_i} }}}}\right)^{{\hbar ^\gamma }_{\intercal}}} \le \mathop {\tilde \prod}\limits _{ \intercal = 1}^{ m}{\left(\mathop {\tilde \prod}\limits _{ i = 1}^{ n}{(1 - \{ \mu _{i{\intercal}}^{\it q}\} )^{{{{{\aleph}^{\it\ell}}_i} \over {\sum\nolimits_{i = 1}^n {{{\aleph}^{\it\ell}}_i} }}}}\right)^{{\hbar ^\gamma }_{\intercal}}} \cr  &  \le \mathop {\tilde \prod}\limits _{ \intercal = 1}^{ m}{\left(\mathop {\tilde \prod}\limits _{ i = 1}^{ n}(1 - {\mathrm{mi}}{{\mathrm{n}}_{\intercal}}\;{\mathrm{mi}}{{\mathrm{n}}_i}\{ \mu _{i{\intercal}}^{\it q}\} )\right)^{{\hbar ^\gamma }_{\intercal}}} \quad  \Leftrightarrow {\left({(1 - {\mathrm{ma}}{{\mathrm{x}}_{\intercal}}\;{\mathrm{ma}}{{\mathrm{x}}_i}\{ \mu _{i{\intercal}}^{\it q}\} )^{\sum\nolimits_{i = 1}^n {{{{{\aleph}^{\it\ell}}_i} \over {\sum\nolimits_{i = 1}^n {{{\aleph}^{\it\ell}}_i} }}} }}\right)^{\sum\nolimits_{\intercal = 1}^m {{\hbar ^\gamma }_{\intercal}} }} \cr &  \le \mathop {\tilde \prod}\limits _{ \intercal = 1}^{ m}{\left(\mathop {\tilde \prod}\limits _{ i = 1}^{ n}{(1 - \mu _{i{\intercal}}^{\it q})^{{{{{\aleph}^{\it\ell}}_i} \over {\sum\nolimits_{i = 1}^n {{{\aleph}^{\it\ell}}_i} }}}}\right)^{{\hbar ^\gamma }_{\intercal}}}  \le {\left({(1 - {\mathrm{mi}}{{\mathrm{n}}_{\intercal}}\;{\mathrm{mi}}{{\mathrm{n}}_i}\{ \mu _{i{\intercal}}^{\it q}\} )^{\sum\nolimits_{i = 1}^n {{{{{\aleph}^{\it\ell}}_i} \over {\sum\nolimits_{i = 1}^n {{{\aleph}^{\it\ell}}_i} }}} }}\right)^{\sum\nolimits_{\intercal = 1}^m {{\hbar ^\gamma }_{\intercal}} }} \cr  &  \Leftrightarrow \left( {1 - {\mathrm{ma}}{{\mathrm{x}}_{\intercal}}\;{\mathrm{ma}}{{\mathrm{x}}_i}\{ \mu _{i{\intercal}}^{\it q}\} } \right) \le \mathop {\tilde \prod}\limits _{ \intercal = 1}^{ m}{\left(\mathop {\tilde \prod}\limits _{ i = 1}^{ n}{(1 - \mu _{i{\intercal}}^{\it q})^{{{{{\aleph}^{\it\ell}}_i} \over {\sum\nolimits_{i = 1}^n {{{\aleph}^{\it\ell}}_i} }}}}\right)^{{\hbar ^\gamma }_{\intercal}}} \cr  &  \le (1 - {\mathrm{mi}}{{\mathrm{n}}_{\intercal}}\;{\mathrm{mi}}{{\mathrm{n}}_i}\{ \mu _{i{\intercal}}^{\it q}\} )  \Leftrightarrow 1 - (1 - {\mathrm{mi}}{{\mathrm{n}}_{\intercal}}\;{\mathrm{mi}}{{\mathrm{n}}_i}\{ \mu _{i{\intercal}}^{\it q}\} ) \le 1 - \mathop {\tilde \prod}\limits _{ \intercal = 1}^{ m}{\left(\mathop {\tilde \prod}\limits _{ i = 1}^{ n}{(1 - \mu _{i{\intercal}}^{\it q})^{{{{{\aleph}^{\it\ell}}_i} \over {\sum\nolimits_{i = 1}^n {{{\aleph}^{\it\ell}}_i} }}}}\right)^{{\hbar ^\gamma }_{\intercal}}}\cr  &  \le 1 - (1 - {\mathrm{ma}}{{\mathrm{x}}_{\intercal}}\;{\mathrm{ma}}{{\mathrm{x}}_i}\{ \mu _{i{\intercal}}^{\it q}\} ) \cr {\mathrm{mi}}{{\mathrm{n}}_{\intercal}}\;{\mathrm{mi}}{{\mathrm{n}}_i}{\{ {\mu _{i{\intercal}}}\} ^{\it q}} & \le \root q \of {1 - \widetilde \prod _{\intercal = 1}^{m}{{\left(\widetilde \prod _{i = 1}^{n}{{(1 - \mu _{i{\intercal}}^{\it q})}^{{{{{\aleph}^{\it\ell}}_i} \over {\sum\nolimits_{i = 1}^n {{{\aleph}^{\it\ell}}_i} }}}}\right)}^{{\hbar ^\gamma }_{\intercal}}}} \cr  &  \le {\mathrm{ma}}{{\mathrm{x}}_{\intercal}}\;{\mathrm{ma}}{{\mathrm{x}}_i}\{ {\mu _{i{\intercal}}}\} }$$

Next for each $i = 1,2,...,n$ and $\intercal = 1,2,...,\it m$, we have

$$\eqalign{{\mathrm{mi}}{{\mathrm{n}}_{\intercal}}\;{\mathrm{mi}}{{\mathrm{n}}_i}\{ {\nu _{i{\intercal}}}\}  &  \le {\nu _{i{\intercal}}} \le {\mathrm{ma}}{{\mathrm{x}}_{\intercal}}\;{\mathrm{ma}}{{\mathrm{x}}_i}\{ {\nu _{i{\intercal}}}\} \\  &  \mathop {\tilde \prod }\limits_{\intercal = 1}^m {\left(\mathop {\tilde \prod }\limits_{i = 1}^n{(mi{n_{\intercal}}\;{\mathrm{mi}}{{\mathrm{n}}_i}\{ {\nu _{i{\intercal}}}\} )^{{{{{\aleph}^{\it\ell}}_i} \over {\sum\nolimits_{i = 1}^n {{{\aleph}^{\it\ell}}_i} }}}}\right)^{{\hbar ^\gamma }_{\intercal}}} \le \mathop {\tilde \prod }\limits_{\intercal = 1}^m{\left(\mathop {\tilde \prod }\limits_{i = 1}^n{(\nu _{i{\intercal}}^{\it q})^{{{{{\aleph}^{\it\ell}}_i} \over {\sum\nolimits_{i = 1}^n {{{\aleph}^{\it\ell}}_i} }}}}\right)^{{\hbar ^\gamma }_{\intercal}}}\\  &  \le \mathop {\tilde \prod }\limits_{\intercal = 1}^m{\left(\mathop {\tilde \prod }\limits_{i = 1}^n{({\mathrm{ma}}{{\mathrm{x}}_{\intercal}}\;{\mathrm{ma}}{{\mathrm{x}}_i}\{ {\nu _{i{\intercal}}}\} )^{{{{{\aleph}^{\it\ell}}_i} \over {\sum\nolimits_{i = 1}^n {{{\aleph}^{\it\ell}}_i} }}}}\right)^{{\hbar ^\gamma }_{\intercal}}} \Leftrightarrow {\left({({\mathrm{mi}}{{\mathrm{n}}_{\intercal}}\;{\mathrm{mi}}{{\mathrm{n}}_i}\{ {\nu _{i{\intercal}}}\} )^{\sum\nolimits_{i = 1}^n {{{{{\aleph}^{\it\ell}}_i} \over {\sum\nolimits_{i = 1}^n {{{\aleph}^{\it\ell}}_i} }}} }}\right)^{\sum\nolimits_{\intercal = 1}^m {{\hbar ^\gamma }_{\intercal}} }}\\  &  \le \mathop {\tilde \prod }\limits_{\intercal = 1}^m{\left(\mathop {\tilde \prod }\limits_{i = 1}^n{({\nu _{i{\intercal}}})^{\sum\nolimits_{i = 1}^n {{{{{\aleph}^{\it\ell}}_i} \over {\sum\nolimits_{i = 1}^n {{{\aleph}^{\it\ell}}_i} }}} }}\right)^{\sum\nolimits_{\intercal = 1}^m {{\hbar ^\gamma }_{\intercal}} }} \le {\left({({\mathrm{ma}}{{\mathrm{x}}_{\intercal}}\;{\mathrm{ma}}{{\mathrm{x}}_i}\{ {\nu _{i{\intercal}}}\} )^{\sum\nolimits_{i = 1}^n {{{{{\aleph}^{\it\ell}}_i} \over {\sum\nolimits_{i = 1}^n {{{\aleph}^{\it\ell}}_i} }}} }}\right)^{\sum\nolimits_{\intercal = 1}^m {{\hbar ^\gamma }_{\intercal}} }}}$$

this implies that

$${\mathrm{mi}}{{\mathrm{n}}_{\intercal}}\;{\mathrm{mi}}{{\mathrm{n}}_i}\{ {\nu _{i{\intercal}}}\} \le \mathop {\tilde \prod} _{ \intercal = 1}^{ m}{\left(\mathop {\tilde \prod} _{ i = 1}^{ n}{({\nu _{i{\intercal}}})^{\sum\nolimits_{i = 1}^n {{{{{\aleph}^{\it\ell}}_i} \over {\sum\nolimits_{i = 1}^n {{{\aleph}^{\it\ell}}_i} }}} }}\right)^{\sum\nolimits_{\intercal = 1}^m {{\hbar ^\gamma }_{\intercal}} }} \le {\mathrm{ma}}{{\mathrm{x}}_{\intercal}}\;{\mathrm{ma}}{{\mathrm{x}}_i}\{ {\nu _{i{\intercal}}}\}$$

As a result of Eqs. (3) and (4), we obtain

$${\mathrm{mi}}{{\mathrm{n}}_{\intercal}}\;{\mathrm{mi}}{{\mathrm{n}}_i}\{ {\mu _{i{\intercal}}}\} \le \root q \of {1 - \widetilde \prod _{\intercal = 1}^{m}{{\left(\widetilde \prod _{i = 1}^{n}{{(1 - \mu _{i{\intercal}}^{\it q})}^{{{{{\aleph}^{\it\ell}}_i} \over {\sum\nolimits_{i = 1}^n {{{\aleph}^{\it\ell}}_i} }}}}\right)}^{{\hbar ^\gamma }_{\intercal}}}} \le {\mathrm{ma}}{{\mathrm{x}}_{\intercal}}\;{\mathrm{ma}}{{\mathrm{x}}_i}\{ {\mu _{i{\intercal}}}\}$$

and

$${\mathrm{mi}}{{\mathrm{n}}_{\intercal}}\;{\mathrm{mi}}{{\mathrm{n}}_i}\{ {\nu _{i{\intercal}}}\} \le \mathop {\tilde \prod }\limits_{\intercal = 1}^m {\left(\mathop {\tilde \prod }\limits_{i = 1}^n {(\nu _{i{\intercal}}^{\it q})^{{{{{\aleph}^{\it\ell}}_i} \over {\sum\nolimits_{i = 1}^n {{{\aleph}^{\it\ell}}_i} }}}}\right)^{{\hbar ^\gamma }_{\intercal}}} \le {\mathrm{ma}}{{\mathrm{x}}_{\intercal}}\;{\mathrm{ma}}{{\mathrm{x}}_i}\{ {\nu _{i{\intercal}}}\}$$

Let ${\aleph^{\ell}} = q \!\! -\!\!  ROFSPWA\left( {{{\mathcal N}^{\beth}}_{{ {\eth}}_{11}},{{\mathcal N}^{\beth}}_{{ {\eth}}_{12}}, \ldots {{\mathcal N}^{\beth}}_{{ {\eth}}_{nm}}} \right) = ({\mu _{{{\aleph}^{\it\ell}}}},{\nu _{{{\aleph}^{\it\ell}}}})$ then by SF given in Definition (3.3), we have

$$\eqalign{S({\aleph^{\ell}})   &  =  \mu _{{{\aleph}^{\it\ell}}}^{\it q} - \nu _{{{\aleph}^{\it\ell}}}^{\it q} + \left({{{e^{\mu _{{{\aleph}^{\it\ell}}}^{\it q} - \nu _{{{\aleph}^{\it\ell}}}^{\it q}}}} \over {{e^{\mu _{{{\aleph}^{\it\ell}}}^{\it q} - \nu _{{{\aleph}^{\it\ell}}}^{\it q}}} + 1}} - {1 \over 2}\right)\pi _{{{\aleph}^{\it\ell}}}^{\it q} \le {({\mathrm{ma}}{{\mathrm{x}}_{\intercal}}{\mathrm{ma}}{{\mathrm{x}}_i}\{ {\mu _{i{\intercal}}}\} )^{\it q}} - {({\mathrm{mi}}{{\mathrm{n}}_{\intercal}}{\mathrm{mi}}{{\mathrm{n}}_i}\{ {\mu _{i{\intercal}}}\} )^{\it q}}\\  &  + \left( {{{{e^{{{({\mathrm{ma}}{{\mathrm{x}}_{\intercal}}{\mathrm{ma}}{{\mathrm{x}}_i}\{ {\mu _{i{\intercal}}}\} )}^{\it q}} - {{({\mathrm{mi}}{{\mathrm{n}}_{\intercal}}{\mathrm{mi}}{{\mathrm{n}}_i}\{ {\mu _{i{\intercal}}}\} )}^{\it q}}}}} \over {{e^{{{({\mathrm{ma}}{{\mathrm{x}}_{\intercal}}{\mathrm{ma}}{{\mathrm{x}}_i}\{ {\mu _{i{\intercal}}}\} )}^{\it q}} - {{({\mathrm{mi}}{{\mathrm{n}}_{\intercal}}{\mathrm{mi}}{{\mathrm{n}}_i}\{ {\mu _{i{\intercal}}}\} )}^{\it q}}}} + 1}} - {1 \over 2}} \right)\pi _{{{\mathcal N}^{\beth}}_{{ {\eth}}_{i{\intercal}}}^ + }^{\it q} = S\left({{\mathcal N}^{\beth}}_{{ {\eth}}_{i{\intercal}}}^ + \right)}$$

This implies $S({\aleph^{\ell}}) \le S\left({{\mathcal N}^{\beth}}_{{ {\eth}}_{i{\intercal}}}^ + \right)$ and

$$\eqalign{S({\aleph^{\ell}})   &  =  \mu _{{{\aleph}^{\it\ell}}}^{\it q} - \nu _{{{\aleph}^{\it\ell}}}^{\it q} + \left({{{e^{\mu _{{{\aleph}^{\it\ell}}}^{\it q} - \nu _{{{\aleph}^{\it\ell}}}^{\it q}}}} \over {{e^{\mu _{{{\aleph}^{\it\ell}}}^{\it q} - \nu _{{{\aleph}^{\it\ell}}}^{\it q}}} + 1}} - {1 \over 2}\right)\pi _{{{\aleph}^{\it\ell}}}^{\it q} \ge {({\mathrm{mi}}{{\mathrm{n}}_{\intercal}}{\mathrm{mi}}{{\mathrm{n}}_i}\{ {\mu _{i{\intercal}}}\} )^{\it q}} - {({\mathrm{ma}}{{\mathrm{x}}_{\intercal}}{\mathrm{ma}}{{\mathrm{x}}_i}\{ {\mu _{i{\intercal}}}\} )^{\it q}}\\  &  + \left( {{{{e^{{{({\mathrm{mi}}{{\mathrm{n}}_{\intercal}}{\mathrm{mi}}{{\mathrm{n}}_i}\{ {\mu _{i{\intercal}}}\} )}^{\it q}} - {{({\mathrm{ma}}{{\mathrm{x}}_{\intercal}}{\mathrm{ma}}{{\mathrm{x}}_i}\{ {\mu _{i{\intercal}}}\} )}^{\it q}}}}} \over {{e^{{{({\mathrm{mi}}{{\mathrm{n}}_{\intercal}}{\mathrm{mi}}{{\mathrm{n}}_i}\{ {\mu _{i{\intercal}}}\} )}^{\it q}} - {{({\mathrm{ma}}{{\mathrm{x}}_{\intercal}}{\mathrm{ma}}{{\mathrm{x}}_i}\{ {\mu _{i{\intercal}}}\} )}^{\it q}}}} + 1}} - {1 \over 2}} \right)\pi _{{{\mathcal N}^{\beth}}_{{ {\eth}}_{i{\intercal}}}^ - }^{\it q} = S\left({{\mathcal N}^{\beth}}_{{ {\eth}}_{i{\intercal}}}^ - \right)}$$

This implies $S({\aleph^{\ell}}) \ge S({{\mathcal N}^{\beth}}_{{ {\eth}}_{i{\intercal}}}^ - )$ and

Consider the following cases,

**Case i:** If $S({\aleph^{\ell}})\lt S({{\mathcal N}^{\beth}}_{{ {\eth}}_{i{\intercal}}}^ + )$ and $S({\aleph^{\ell}}) \gt S({{\mathcal N}^{\beth}}_{{ {\eth}}_{i{\intercal}}}^ - )$, by the comparison of two q-ROFSNs, we get

$${{\mathcal N}^{\beth}}_{{ {\eth}}_{i{\intercal}}}^ - \le q \!\! -\!\!  ROFSPWA({{\mathcal N}^{\beth}}_{{ {\eth}}_{11}},{{\mathcal N}^{\beth}}_{{ {\eth}}_{12}},{{\mathcal N}^{\beth}}_{{ {\eth}}_{13}},...,{{\mathcal N}^{\beth}}_{{ {\eth}}_{nm}}) \le {{\mathcal N}^{\beth}}_{{ {\eth}}_{i{\intercal}}}^ +$$

**Case ii:** If $S({\aleph^{\ell}}) = S({{\mathcal N}^{\beth}}_{{ {\eth}}_{i{\intercal}}}^ + )$, that is

$$\eqalign{\mu _{{{\aleph}^{\it\ell}}}^{\it q} - \nu _{{{\aleph}^{\it\ell}}}^{\it q} + \left( {{{{e^{\mu _{{{\aleph}^{\it\ell}}}^{\it q} - \nu _{{{\aleph}^{\it\ell}}}^{\it q}}}} \over {{e^{\mu _{{{\aleph}^{\it\ell}}}^{\it q} - \nu _{{{\aleph}^{\it\ell}}}^{\it q}}} + 1}} - {1 \over 2}} \right)\pi _{{{\aleph}^{\it\ell}}}^{\it q}  &  = {({\mathrm{ma}}{{\mathrm{x}}_{\intercal}}{\mathrm{ma}}{{\mathrm{x}}_i}\{ {\mu _{i{\intercal}}}\} )^{\it q}} - {({\mathrm{mi}}{{\mathrm{n}}_{\intercal}}{\mathrm{mi}}{{\mathrm{n}}_i}\{ {\mu _{i{\intercal}}}\} )^{\it q}}\\  &  + \left( {{{{e^{{{({\mathrm{ma}}{{\mathrm{x}}_{\intercal}}{\mathrm{ma}}{{\mathrm{x}}_i}\{ {\mu _{i{\intercal}}}\} )}^{\it q}} - {{({\mathrm{mi}}{{\mathrm{n}}_{\intercal}}{\mathrm{mi}}{{\mathrm{n}}_i}\{ {\mu _{i{\intercal}}}\} )}^{\it q}}}}} \over {{e^{{{({\mathrm{ma}}{{\mathrm{x}}_{\intercal}}{\mathrm{ma}}{{\mathrm{x}}_i}\{ {\mu _{i{\intercal}}}\} )}^{\it q}} - {{({\mathrm{mi}}{{\mathrm{n}}_{\intercal}}{\mathrm{mi}}{{\mathrm{n}}_i}\{ {\mu _{i{\intercal}}}\} )}^{\it q}}}} + 1}} - {1 \over 2}} \right)\pi _{{{\mathcal N}^{\beth}}_{{ {\eth}}_{i{\intercal}}}^ + }^{\it q}}$$

Then by using the above inequalities, we get

$${\mu _{{{\aleph}^{\it\ell}}}} = {\mathrm{ma}}{{\mathrm{x}}_{\intercal}}{\mathrm{ma}}{{\mathrm{x}}_i}\{ {\mu _{i{\intercal}}}\} \quad and \quad{\nu _{{{\aleph}^{\it\ell}}}} = {\mathrm{mi}}{{\mathrm{n}}_{\intercal}}{\mathrm{mi}}{{\mathrm{n}}_i}\{ {\nu _{i{\intercal}}}\} .\quad{\mathrm{Thus}}\quad\pi _{{{\aleph}^{\it\ell}}}^{\it q} = \pi _{{{\mathcal N}^{\beth}}_{{ {\eth}}_{i{\intercal}}}^ + }^{\it q}$$

Hence by comparison of two q-ROFSNs, we have

$$q \!\! -\!\!  ROFSPWA\left( {{{\mathcal N}^{\beth}}_{{ {\eth}}_{11}},{{\mathcal N}^{\beth}}_{{ {\eth}}_{12}},{{\mathcal N}^{\beth}}_{{ {\eth}}_{13}},...,{{\mathcal N}^{\beth}}_{{ {\eth}}_{nm}}} \right) \le {{\mathcal N}^{\beth}}_{{ {\eth}}_{i{\intercal}}}^ +$$

**Case iii:** If $S({\aleph^{\ell}}) = S\left({{\mathcal N}^{\beth}}_{{ {\eth}}_{i{\intercal}}}^ - \right)$, that is

$$\eqalign{\mu _{{{\aleph}^{\it\ell}}}^{\it q} - \nu _{{{\aleph}^{\it\ell}}}^{\it q} + \left( {{{{e^{\mu _{{{\aleph}^{\it\ell}}}^{\it q} - \nu _{{{\aleph}^{\it\ell}}}^{\it q}}}} \over {{e^{\mu _{{{\aleph}^{\it\ell}}}^{\it q} - \nu _{{{\aleph}^{\it\ell}}}^{\it q}}} + 1}} - {1 \over 2}} \right)\pi _{{{\aleph}^{\it\ell}}}^{\it q}  &  = {({\mathrm{mi}}{{\mathrm{n}}_{\intercal}}{\mathrm{mi}}{{\mathrm{n}}_i}\{ {\mu _{i{\intercal}}}\} )^{\it q}} - {({\mathrm{ma}}{{\mathrm{x}}_{\intercal}}{\mathrm{ma}}{{\mathrm{x}}_i}\{ {\mu _{i{\intercal}}}\} )^{\it q}}\\  &  + \left( {{{{e^{{{\left( {{\mathrm{mi}}{{\mathrm{n}}_{\intercal}}{\mathrm{mi}}{{\mathrm{n}}_i}\{ {\mu _{i{\intercal}}}\} } \right)}^{\it q}} - {{({\mathrm{ma}}{{\mathrm{x}}_{\intercal}}{\mathrm{ma}}{{\mathrm{x}}_i}\{ {\mu _{i{\intercal}}}\} )}^{\it q}}}}} \over {{e^{{{({\mathrm{mi}}{{\mathrm{n}}_{\intercal}}{\mathrm{mi}}{{\mathrm{n}}_i}\{ {\mu _{i{\intercal}}}\} )}^{\it q}} - {{({\mathrm{ma}}{{\mathrm{x}}_{\intercal}}{\mathrm{ma}}{{\mathrm{x}}_i}\{ {\mu _{i{\intercal}}}\} )}^{\it q}}}} + 1}} - {1 \over 2}} \right)\pi _{{{\mathcal N}^{\beth}}_{{ {\eth}}_{i{\intercal}}}^ - }^{\it q}}$$

Then by using the above inequalities, we get

$${\mu _{{{\aleph}^{\it\ell}}}} = {\mathrm{mi}}{{\mathrm{n}}_{\intercal}}{\mathrm{mi}}{{\mathrm{n}}_i}\{ {\mu _{i{\intercal}}}\} \quad{\mathrm{and}}\quad{\nu _{{{\aleph}^{\it\ell}}}} = {\mathrm{ma}}{{\mathrm{x}}_{\intercal}}{\mathrm{ma}}{{\mathrm{x}}_i}\{ {\nu _{i{\intercal}}}\} .\quad{\mathrm{Thus}}\quad\pi _{{{\aleph}^{\it\ell}}}^{\it q} = \pi _{{{\mathcal N}^{\beth}}_{{ {\eth}}_{i{\intercal}}}^ - }^{\it q}$$

This implies

$$q \!\! -\!\!  ROFSPWA({{\mathcal N}^{\beth}}_{{ {\eth}}_{11}},{{\mathcal N}^{\beth}}_{{ {\eth}}_{12}},{{\mathcal N}^{\beth}}_{{ {\eth}}_{13}},...,{{\mathcal N}^{\beth}}_{{ {\eth}}_{nm}}) \le {{\mathcal N}^{\beth}}_{{ {\eth}}_{i{\intercal}}}^ -$$

Hence, it is proved that

$${{\mathcal N}^{\beth}}_{{ {\eth}}_{i{\intercal}}}^ {-} \leqq- ROFSPWA({{\mathcal N}^{\beth}}_{{ {\eth}}_{11}},{{\mathcal N}^{\beth}}_{{ {\eth}}_{12}},{{\mathcal N}^{\beth}}_{{ {\eth}}_{13}},...,{{\mathcal N}^{\beth}}_{{ {\eth}}_{nm}}) \le {{\mathcal N}^{\beth}}_{{ {\eth}}_{i{\intercal}}}^ +$$

(**Monotonicity**): Since ${\mu _{i{\intercal}}} \le {p_{i{\intercal}}}$ and ${\nu _{i{\intercal}}} \ge {r_{i{\intercal}}}$, (i = 1, 2,…, n) and $(\intercal = 1,2,...,\it m)$, then this implies that to

$$\eqalign{{\mu _{i{\intercal}}} \le {p_{i{\intercal}}} \le 1 \Rightarrow 1 - {p_{i{\intercal}}} \le 1 - {\mu _{i{\intercal}}} \Rightarrow 1 - p_{i{\intercal}}^{\it q} \le 1 - \mu _{i{\intercal}}^{\it q}}$$

$$\eqalign{  &  \Rightarrow \mathop {\tilde \prod }\limits_{\intercal = 1}^m {\left( {\mathop {\tilde \prod }\limits_{i = 1}^n {{(1 - p_{i{\intercal}}^{\it q})}^{{{{{\aleph}^{\it\ell}}_i} \over {\sum\nolimits_{i = 1}^n {{{\aleph}^{\it\ell}}_i} }}}}} \right)^{{\hbar ^\gamma }_{\intercal}}} \le \mathop {\tilde \prod }\limits_{\intercal = 1}^m {\left( {\mathop {\tilde \prod }\limits_{i = 1}^n {{(1 - \mu _{i{\intercal}}^{\it q})}^{{{{{\aleph}^{\it\ell}}_i} \over {\sum\nolimits_{i = 1}^n {{{\aleph}^{\it\ell}}_i} }}}}} \right)^{{\hbar ^\gamma }_{\intercal}}}\\  &  \Rightarrow 1 - \mathop {\tilde \prod }\limits_{\intercal = 1}^m {\left( {\mathop {\tilde \prod }\limits_{i = 1}^n {{(1 - \mu _{i{\intercal}}^{\it q})}^{{{{{\aleph}^{\it\ell}}_i} \over {\sum\nolimits_{i = 1}^n {{{\aleph}^{\it\ell}}_i} }}}}} \right)^{{\hbar ^\gamma }_{\intercal}}}\\  &  \le 1 - \mathop {\tilde \prod }\limits_{\intercal = 1}^m {\left( {\mathop {\tilde \prod }\limits_{i = 1}^n {{(1 - p_{i{\intercal}}^{\it q})}^{{{{{\aleph}^{\it\ell}}_i} \over {\sum\nolimits_{i = 1}^n {{{\aleph}^{\it\ell}}_i} }}}}} \right)^{{\hbar ^\gamma }_{\intercal}}}\root q \of {1 - \widetilde \prod _{\intercal = 1}^{m}{{\left(\widetilde \prod _{i = 1}^{n}{{(1 - \mu _{i{\intercal}}^{\it q})}^{{{{{\aleph}^{\it\ell}}_i} \over {\sum\nolimits_{i = 1}^n {{{\aleph}^{\it\ell}}_i} }}}}\right)}^{{\hbar ^\gamma }_{\intercal}}}} \\  &  \le \root q \of {1 - \widetilde \prod _{\intercal = 1}^{m}{{\left(\widetilde \prod _{i = 1}^{n}{{(1 - \mu _{i{\intercal}}^{\it q})}^{{{{{\aleph}^{\it\ell}}_i} \over {\sum\nolimits_{i = 1}^n {{{\aleph}^{\it\ell}}_i} }}}}\right)}^{{\hbar ^\gamma }_{\intercal}}}} {.\,\,\rm Furthermore,}}$$

$$\eqalign{{\nu _{i{\intercal}}} \ge {r_{i{\intercal}}} &   \Rightarrow \left(\mathop {\tilde \prod }\limits_{i = 1}^n{({\nu _{i{\intercal}}})^{{{{{\aleph}^{\it\ell}}_i} \over {\sum\nolimits_{i = 1}^n {{{\aleph}^{\it\ell}}_i} }}}}\right) \ge \mathop {\tilde \prod }\limits_{i = 1}^n{({\nu _{i{\intercal}}})^{{{{{\aleph}^{\it\ell}}_i} \over {\sum\nolimits_{i = 1}^n {{{\aleph}^{\it\ell}}_i} }}}}\\  &  \Rightarrow \mathop {\tilde \prod }\limits_{\intercal = 1}^m{\left(\mathop {\tilde \prod }\limits_{i = 1}^n{({\nu _{i{\intercal}}})^{{{{{\aleph}^{\it\ell}}_i} \over {\sum\nolimits_{i = 1}^n {{{\aleph}^{\it\ell}}_i} }}}}\right)^{{\hbar ^\gamma }_{\intercal}}} \ge \mathop {\tilde \prod }\limits_{\intercal = 1}^m{\left(\mathop {\tilde \prod }\limits_{i = 1}^n{({r_{i{\intercal}}})^{{{{{\aleph}^{\it\ell}}_i} \over {\sum\nolimits_{i = 1}^n {{{\aleph}^{\it\ell}}_i} }}}}\right)^{{\hbar ^\gamma }_{\intercal}}}}$$

Let

$$\eqalign{{\aleph^{\ell}}_{{{\mathcal N}^{\beth}}}  &  = q \!\! -\!\!  ROFSPWA\left( {{{\mathcal N}^{\beth}}_{{ {\eth}}_{11}},{{\mathcal N}^{\beth}}_{{ {\eth}}_{12}},{{\mathcal N}^{\beth}}_{{ {\eth}}_{13}},...,{{\mathcal N}^{\beth}}_{{ {\eth}}_{nm}}} \right) = ({\mu _{{{\aleph}^{\it\ell}}_{{{\mathcal N}^{\beth}}}}},{\nu _{{{\aleph}^{\it\ell}}_{{{\mathcal N}^{\beth}}}}}) \\ {\aleph^{\ell}}_\Gamma  &  = q \!\! -\!\!  ROFSPWA\left( {{\Gamma _{{ {\eth}}_{11}}},{{\mathcal N}^{\beth}}_{{ {\eth}}_{12}},{{\mathcal N}^{\beth}}_{{ {\eth}}_{13}},...,{{\mathcal N}^{\beth}}_{{ {\eth}}_{nm}}} \right) = ({\mu _{{{\aleph}^{\it\ell}}_{{{\mathcal N}^{\beth}}}}},{\nu _{{{\aleph}^{\it\ell}}_{{{\mathcal N}^{\beth}}}}})}$$

From Eqs. (5) and 6, we have

$${\mu _{{{\aleph}^{\it\ell}}_{{{\mathcal N}^{\beth}}}}} \le {p_{{{\aleph}^{\it\ell}}_\Gamma }}\quad and \quad{\nu _{{{\aleph}^{\it\ell}}_{{{\mathcal N}^{\beth}}}}} \ge {p_{{{\aleph}^{\it\ell}}_\Gamma }}$$

then by SF given in Definition 9, we have

$$S({\aleph^{\ell}}_{{{\mathcal N}^{\beth}}}) \le S({\aleph^{\ell}}_\Gamma )$$

In view of that direction, consider the following cases,

**Case i:** If $S({\aleph^{\ell}}_{{{\mathcal N}^{\beth}}})\lt S({\aleph^{\ell}}_\Gamma )$ and $S({\aleph^{\ell}}) \gt S({{\mathcal N}^{\beth}}_{{ {\eth}}_{i{\intercal}}}^ - )$, by the comparison of two q-ROFSNs, we get

$$q \!\! -\!\!  ROFSPWA\left( {{{\mathcal N}^{\beth}}_{{ {\eth}}_{11}},{{\mathcal N}^{\beth}}_{{ {\eth}}_{12}},{{\mathcal N}^{\beth}}_{{ {\eth}}_{13}},...,{{\mathcal N}^{\beth}}_{{ {\eth}}_{nm}}} \right) \le q \!\! -\!\!  ROFSPWA({\Gamma _{{ {\eth}}_{11}}},{\Gamma _{{ {\eth}}_{12}}},{\Gamma _{{ {\eth}}_{13}}},...,{\Gamma _{{ {\eth}}_{nm}}})$$

**Case ii:** If $S({\aleph^{\ell}}_{{{\mathcal N}^{\beth}}})\lt S({\aleph^{\ell}}_\Gamma )$, that is

$$S({\aleph^{\ell}}_{{{\mathcal N}^{\beth}}}) = \mu _{{{\aleph}^{\it\ell}}_\Gamma }^{\it q} - \nu _{{{\aleph}^{\it\ell}}_\Gamma }^{\it q} + \left({{{e^{\mu _{{{\aleph}^{\it\ell}}_\Gamma }^{\it q} - \nu _{{{\aleph}^{\it\ell}}_\Gamma }^{\it q}}}} \over {{e^{\mu _{{{\aleph}^{\it\ell}}_\Gamma }^{\it q} - \nu _{{{\aleph}^{\it\ell}}_\Gamma }^{\it q}}} + 1}} - {1 \over 2}\right)\pi _{{{\aleph}^{\it\ell}}_\Gamma }^{\it q} = = \mu _{{{\aleph}^{\it\ell}}_\Gamma }^{\it q} - \nu _{{{\aleph}^{\it\ell}}_\Gamma }^{\it q} + \left({{{e^{\mu _{{{\aleph}^{\it\ell}}_\Gamma }^{\it q} - \nu _{{{\aleph}^{\it\ell}}_\Gamma }^{\it q}}}} \over {{e^{\mu _{{{\aleph}^{\it\ell}}_\Gamma }^{\it q} - \nu _{{{\aleph}^{\it\ell}}_\Gamma }^{\it q}}} + 1}} - {1 \over 2}\right)\pi _{{{\aleph}^{\it\ell}}_\Gamma }^{\it q} = S({\aleph^{\ell}}_{{{\mathcal N}^{\beth}}})$$

then by above inequality, we have

$${\mu _{{{\aleph}^{\it\ell}}_\Gamma }} = {p_{{{\aleph}^{\it\ell}}_\Gamma }}\quad and\quad {\nu _{{{\aleph}^{\it\ell}}_\Gamma }} = {r_{{{\aleph}^{\it\ell}}_\Gamma }}$$

Hence

$$\pi _{{{\aleph}^{\it\ell}}_\Gamma }^{\it q} = \pi _{{{\aleph}^{\it\ell}}_\Gamma }^{\it q} \Rightarrow ({\mu _{{{\aleph}^{\it\ell}}_\Gamma }},{\nu _{{{\aleph}^{\it\ell}}_\Gamma }}) = ({p_{{{\aleph}^{\it\ell}}_\Gamma }},{r_{{{\aleph}^{\it\ell}}_\Gamma }})$$

Therefore, it is proved that

$$q \!\! -\!\!  ROFSPWA({{\mathcal N}^{\beth}}_{{ {\eth}}_{11}},{{\mathcal N}^{\beth}}_{{ {\eth}}_{12}},{{\mathcal N}^{\beth}}_{{ {\eth}}_{13}},...,{{\mathcal N}^{\beth}}_{{ {\eth}}_{nm}}) \le q \!\! -\!\!  ROFSPWA({\Gamma _{{ {\eth}}_{11}}},{\Gamma _{{ {\eth}}_{12}}},{\Gamma _{{ {\eth}}_{13}}},...,{\Gamma _{{ {\eth}}_{nm}}})$$

(**Shift Invariance**): Since ${\Gamma _e} = (p,r)$ and ${{\mathcal N}^{\beth}}_{{ {\eth}}_{i{\intercal}}} = ({\mu _{{ {\eth}}_{i{\intercal}}}},{\nu _{{ {\eth}}_{i{\intercal}}}})$ are the q-ROFSNs, so

$${{\mathcal N}^{\beth}}_{{ {\eth}}_{11}} \oplus {\Gamma _e} = \left(\root q \of {1 - (1 - \mu _{i{\intercal}}^{\it q})(1 - {p^{\it q}})} ,{\nu _{i{\intercal}}}r\right)$$

Therefore,

$$\eqalign{{\mathrm{q \!\! -\!\!  ROFSWA \quad}} &  ({{\mathcal N}^{\beth}}_{{ {\eth}}_{11}} \oplus {\Gamma _e}, \ldots ,{{\mathcal N}^{\beth}}_{{ {\eth}}_{nm}} \oplus {\Gamma _e})\\  &  = \oplus _{\intercal = 1}^m{\hbar ^\gamma }_{\intercal}\left( \oplus _{i = 1}^n{{{{\aleph}^{\it\ell}}_i} \over {\sum\nolimits_{i = 1}^n {{{\aleph}^{\it\ell}}_i} }}({{\mathcal N}^{\beth}}_{{ {\eth}}_{nm}} \oplus {\Gamma _e})\right)\\  &  = \left\langle \root q \of {1 - \widetilde \prod _{\intercal = 1}^{m}{{\left(\widetilde \prod _{i = 1}^{n}{{(1 - \mu _{i{\intercal}}^{\it q})}^{{{{{\aleph}^{\it\ell}}_i} \over {\sum\nolimits_{i = 1}^n {{{\aleph}^{\it\ell}}_i} }}}}{{(1 - {p^{\it q}})}^{{{{{\aleph}^{\it\ell}}_i} \over {\sum\nolimits_{i = 1}^n {{{\aleph}^{\it\ell}}_i} }}}}\right)}^{{\hbar ^\gamma }_{\intercal}}}} ,\mathop{\widetilde \prod} _{ \intercal = 1}^{ m}{\left(\mathop{\widetilde \prod} _{ i = 1}^{ n}\nu _{i{\intercal}}^{{{{{\aleph}^{\it\ell}}_i} \over {\sum\nolimits_{i = 1}^n {{{\aleph}^{\it\ell}}_i} }}}{r^{{{{{\aleph}^{\it\ell}}_i} \over {\sum\nolimits_{i = 1}^n {{{\aleph}^{\it\ell}}_i} }}}}\right)^{{\hbar ^\gamma }_{\intercal}}}\right\rangle \\  &  = \left\langle \root q \of {1 - (1 - {p^{\it q}})\widetilde \prod _{\intercal = 1}^{m}{{\left(\widetilde \prod _{i = 1}^{n}{{(1 - \mu _{i{\intercal}}^{\it q})}^{{{{{\aleph}^{\it\ell}}_i} \over {\sum\nolimits_{i = 1}^n {{{\aleph}^{\it\ell}}_i} }}}}\right)}^{{\hbar ^\gamma }_{\intercal}}}} ,r\mathop{\widetilde \prod} _{ \intercal = 1}^{ m}\boldsymbol(\mathop{\widetilde \prod} _{ i = 1}^{ n}\nu _{i{\intercal}}^{{{{{\aleph}^{\it\ell}}_i} \over {\sum\nolimits_{i = 1}^n {{{\aleph}^{\it\ell}}_i} }}}\right\rangle \\  &  = \left\langle \root q \of {1 - \widetilde \prod _{\intercal = 1}^{m}{{\left(\widetilde \prod _{i = 1}^{n}{{(1 - \mu _{i{\intercal}}^{\it q})}^{{{{{\aleph}^{\it\ell}}_i} \over {\sum\nolimits_{i = 1}^n {{{\aleph}^{\it\ell}}_i} }}}}\right)}^{{\hbar ^\gamma }_{\intercal}}}} ,\mathop{\widetilde \prod} _{ \intercal = 1}^{ m}\boldsymbol(\mathop{\widetilde \prod} _{ i = 1}^{ n}\nu _{i{\intercal}}^{{{{{\aleph}^{\it\ell}}_i} \over {\sum\nolimits_{i = 1}^n {{{\aleph}^{\it\ell}}_i} }}}\right\rangle}$$

Hence the required result is proved.

(**Homogeneity**): Consider $\lambda \ge 0$ be any real number and ${{\mathcal N}^{\beth}}_{{ {\eth}}_{i{\intercal}}} = ({\mu _{i{\intercal}}},{\nu _{i{\intercal}}})$ be a q-ROFSN, then

$$\lambda {{\mathcal N}^{\beth}}_{{ {\eth}}_{i{\intercal}}} = \left\langle \root q \of {1 - (1 - \mu _{i{\intercal}}^{\it q})f{)^{{\hbar ^\gamma }_{}{\intercal}}}} ,\nu i \intercal{^\lambda }\right\rangle$$

Now

$$\eqalign{q - ROFSPWA(\lambda
{{\mathcal N}^{\beth}}_{\eth_{11}},&\lambda {{\mathcal N}^{\beth}}_{\eth_{12}},\lambda {{\mathcal N}^{\beth}}_{\eth_{13}},...,\lambda {{\mathcal N}^{\beth}}_{\eth_{nm}})\cr & = \langle 
\root q \of {1 - \widetilde \prod _{\intercal = 1}^m{{(\widetilde \prod _{i = 1}^n{{(1 - \mu _{i\intercal}^q)}^{\lambda {{{{\aleph}^{\ell}}_i} \over {\sum
\nolimits_{i = 1}^n {{{\aleph}^{\ell}}_i} }}}})}^{{\hbar ^\gamma }_{\intercal}}}} ,\mathop{\widetilde \prod} _{\intercal = 1}^m{(\mathop{\widetilde \prod} _{i = 1}^n
u _{i\intercal}^{\lambda {{{{\aleph}^{\ell}}_i} \over {\sum
\nolimits_{i = 1}^n {{{\aleph}^{\ell}}_i} }}})^{{\hbar ^\gamma }_{\intercal}}}
\rangle \cr & = {\langle 
\root q \of {1 - (\widetilde \prod _{\intercal = 1}^m{{(\widetilde \prod _{i = 1}^n{{(1 - \mu _{i\intercal}^q)}^{{{{{\aleph}^{\ell}}_i} \over {\sum
\nolimits_{i = 1}^n {{{\aleph}^{\ell}}_i} }}}})}^{{\hbar ^\gamma }_{\intercal}}}} )^\lambda },\cr & {(\mathop{\widetilde \prod} _{\intercal = 1}^m{(\mathop{\widetilde \prod} _{i = 1}^n
u _{i\intercal}^{\lambda {{{{\aleph}^{\ell}}_i} \over {\sum
\nolimits_{i = 1}^n {{{\aleph}^{\ell}}_i} }}})^{{\hbar ^\gamma }_{\intercal}}})^\lambda }
\rangle \cr & = \lambda q - ROFSPWA({{\mathcal N}^{\beth}}_{\eth_{11}},{{\mathcal N}^{\beth}}_{\eth_{12}},{{\mathcal N}^{\beth}}_{\eth_{13}},...,{{\mathcal N}^{\beth}}_{\eth_{nm}})}$$

Therefore, the required property is proved.

### q-ROFSPWGA operator

**Definition 4.4.** Assume that ${{\mathcal N}^{\beth}}_{{ {\eth}}_{i{\intercal}}} = \langle {\mu _{i{\intercal}}},{\nu _{i{\intercal}}}\rangle$ for $(i = 1,2,...,n$ and $\intercal = 1,2,...,\it m)$ be a agglomeration of q-ROFSNs, and WVs ${\hbar ^\gamma } = \{ {\hbar ^\gamma }_1,{\hbar ^\gamma }_2, \cdots {\hbar ^\gamma }_n\}$ and ${{{{\aleph}^{\it\ell}}_{\intercal}} \over {\sum\nolimits_{\intercal = 1}^n {{{\aleph}^{\it\ell}}_{\intercal}} }} = \left\{ {{{{{\aleph}^{\it\ell}}_1} \over {\sum\nolimits_{\intercal = 1}^n {{{\aleph}^{\it\ell}}_{\intercal}} }}_1},{{{{{\aleph}^{\it\ell}}_2} \over {\sum\nolimits_{\intercal = 1}^n {{{\aleph}^{\it\ell}}_{\intercal}} }}_2}, \cdots ,{{{{{\aleph}^{\it\ell}}_n} \over {\sum\nolimits_{\intercal = 1}^n {{{\aleph}^{\it\ell}}_{\intercal}} }}_m}\right\}$ for the decision experts ${{\mathcal D}_i}$ and for attributes ${ {\eth}}_{\intercal}^{\prime}$ respectively with the conditions that $\sum\nolimits_{i = 1}^n {{w_i}} = 1$ and $\sum\nolimits_{\intercal = 1}^m {{v_{\intercal}}} = 1$. Then the mapping for $q \!\! -\!\!  ROFSPWG:{\Omega ^n} \to \Omega$, be a n dimension mapping.

(3) $$q \!\! -\!\!  ROFSPWG\left( {{{\mathcal N}^{\beth}}_{{ {\eth}}_{11}}, \ldots {{\mathcal N}^{\beth}}_{{ {\eth}}_{nm}}} \right) = \oplus _{k = 1}^m{\hbar ^\gamma }_{\intercal}\left( {{{{{\aleph}^{\it\ell}}_1} \over {\sum\nolimits_{\intercal = 1}^n {{{\aleph}^{\it\ell}}_{\intercal}} }}{{\mathcal N}^{\beth}}_{{ {\eth}}_{11}} \oplus {{{{\aleph}^{\it\ell}}_2} \over {\sum\nolimits_{\intercal = 1}^n {{{\aleph}^{\it\ell}}_{\intercal}} }}{{\mathcal N}^{\beth}}_{{ {\eth}}_{12}} \oplus \ldots , \oplus {{{{\aleph}^{\it\ell}}_n} \over {\sum\nolimits_{\intercal = 1}^n {{{\aleph}^{\it\ell}}_{\intercal}} }}{{\mathcal N}^{\beth}}_{{ {\eth}}_{nm}}} \right)$$

where ${\aleph^{\ell}}_{\intercal} = \widetilde \prod _{k = 1}^{j - 1}U({{\mathcal N}^{\beth}}_k)$ $(\intercal = 2 \ldots ,\it n)$, ${\aleph^{\ell}}_1 = 1$ and $U({{\mathcal N}^{\beth}}_k)$ is the score of ${k^{th}}$ q-ROFN. We can consider q-ROFSPWG operator by thy theorem below

**Theorem 4.5** Consider that ${{\mathcal N}^{\beth}}_{i{\intercal}} = \langle {\mu _{i{\intercal}}},{\nu _{i{\intercal}}}\rangle$ be a agglomeration of q-ROFSNs, we can find $q \!\! -\!\!  ROFSPWG$ operator by,

(4) $$q  - ROFSPWG({{\mathcal
N}^{\beth}}_{{ {\eth}}_{11}},{{\mathcal N}^{\beth}}_{{ {\eth}}_{12}},
\ldots {{\mathcal N}^{\beth}}_{{ {\eth}}_{nm}}) = \left\langle
\mathop {\tilde {\prod}} _{ \intercal = 1}^{n}{\left(\mathop{\tilde
\prod} _{\intercal = 1}^{n}\mu _{i{\intercal}}^{{{{{\aleph}^{\it\ell}}_{\intercal}} \over {\sum\nolimits_{\intercal = 1}^n {{{\aleph}^{\it\ell}}_{\intercal}} }}}\right)^{{\hbar ^\gamma }_{\intercal}}},\root q \of {1 - \tilde \prod _{\intercal = 1}^{m}{{\left(\tilde \prod _{i = 1}^{n}{{(1 - \nu _{i{\intercal}}^{\it q})}^{{{{{\aleph}^{\it\ell}}_{\intercal}} \over {\sum\nolimits_{\intercal = 1}^n {{{\aleph}^{\it\ell}}_{\intercal}} }}}}\right)}^{{\hbar ^\gamma }_{\intercal}}}} \right\rangle$$

As we know from operation laws, mathematical induction may be used to prove a given result that ${{\mathcal N}^{\beth}}_{{ {\eth}}_{11}} \oplus {{\mathcal N}^{\beth}}_{{ {\eth}}_{12}} = ({\mu _{11}}{\mu _{12}},\root q \of {{{({\nu _{11}})}^{\it q}} + {{({\nu _{12}})}^{\it q}} - {{({\nu _{11}})}^{\it q}}{{({\nu _{12}})}^{\it q}}} )$ and $\lambda {{\mathcal N}^{\beth}} = ({\mu ^{\lambda ,\root q \of {1 - [1 - {\nu ^{\it q}}]} ,}})$ for $\lambda \ge 1$.

First we will show that the Eq. (1) is true for n = 2 and m = 2, so we have

$$\eqalign{   &  q \!\! -\!\!  ROFSPWG({{\mathcal N}^{\beth}}_{{ {\eth}}_{11}},{{\mathcal N}^{\beth}}_{{ {\eth}}_{12}})  \cr    &   =  \oplus _{\intercal = 1}^2{\hbar ^\gamma }_{\intercal}\left( \oplus _{i = 1}^2{{{{\aleph}^{\ell}}_{\intercal}} \over {\sum\nolimits_{\intercal = 1}^n {{{\aleph}^{\ell}}_{\intercal}} }}{{\mathcal N}^{\beth}}_{{ {\eth}}_{i{\intercal}}}\right)  \cr    &   = {\hbar ^\gamma }_1\left( \oplus _{i = 1}^2{{{{\aleph}^{\ell}}_i} \over {\sum\nolimits_{i = 1}^n {{{\aleph}^{\ell}}_i} }}{{\mathcal N}^{\beth}}_{{ {\eth}}_{i1}}\right) \oplus {\hbar ^\gamma }_2\left( \oplus _{i = 1}^2{{{{\aleph}^{\ell}}_i} \over {\sum\nolimits_{i = 1}^n {{{\aleph}^{\ell}}_i} }}{{\mathcal N}^{\beth}}_{{ {\eth}}_{i2}}\right)  \cr    &   = {\hbar ^\gamma }_1\left({{{{\aleph}^{\ell}}_1} \over {\sum\nolimits_{i = 1}^n {{{\aleph}^{\ell}}_i} }}{{\mathcal N}^{\beth}}_{{ {\eth}}_{11}} \oplus {{{{\aleph}^{\ell}}_2} \over {\sum\nolimits_{i = 1}^n {{{\aleph}^{\ell}}_i} }}{{\mathcal N}^{\beth}}_{{ {\eth}}_{21}}\right) \oplus {\hbar ^\gamma }_2\left({{{{\aleph}^{\ell}}_2} \over {\sum\nolimits_{i = 1}^n {{{\aleph}^{\ell}}_i} }}{{\mathcal N}^{\beth}}_{{ {\eth}}_{12}} \oplus {{{{\aleph}^{\ell}}_2} \over {\sum\nolimits_{i = 1}^n {{{\aleph}^{\ell}}_i} }}{{\mathcal N}^{\beth}}_{{ {\eth}}_{22}}\right)  \cr    &   = {\hbar ^\gamma }_1\left\{ \left(\mu _{11}^{{{{{\aleph}^{\ell}}_{\intercal}} \over {\sum\nolimits_{\intercal = 1}^n {{{\aleph}^{\ell}}_{\intercal}} }}},\root q \of {1 - {{(1 - \nu _{11}^{\it q})}^{{{{{\aleph}^{\ell}}_{\intercal}} \over {\sum\nolimits_{\intercal = 1}^n {{{\aleph}^{\ell}}_{\intercal}} }}}}} ,\right) \oplus \left(\mu _{21}^{{{{{\aleph}^{\ell}}_{\intercal}} \over {\sum\nolimits_{\intercal = 1}^n {{{\aleph}^{\ell}}_{\intercal}} }}},\root q \of {1 - {{(1 - \nu _{21}^{\it q})}^{{{{{\aleph}^{\ell}}_{\intercal}} \over {\sum\nolimits_{\intercal = 1}^n {{{\aleph}^{\ell}}_{\intercal}} }}}}} \right)\right\}   \cr    &   \oplus {\hbar ^\gamma }_2\left\{ \left(\mu _{12}^{{{{{\aleph}^{\ell}}_{\intercal}} \over {\sum\nolimits_{\intercal = 1}^n {{{\aleph}^{\ell}}_{\intercal}} }}},\root q \of {1 - {{(1 - \nu _{12}^{\it q})}^{{{{{\aleph}^{\ell}}_{\intercal}} \over {\sum\nolimits_{\intercal = 1}^n {{{\aleph}^{\ell}}_{\intercal}} }}}}} \right) \oplus \left(\mu _{22}^{{{{{\aleph}^{\ell}}_{\intercal}} \over {\sum\nolimits_{\intercal = 1}^n {{{\aleph}^{\ell}}_{\intercal}} }}},\root q \of {1 - {{(1 - \nu _{22}^{\it q})}^{{{{{\aleph}^{\ell}}_{\intercal}} \over {\sum\nolimits_{\intercal = 1}^n {{{\aleph}^{\ell}}_{\intercal}} }}}}} \right)\right\}   \cr    &   = {\hbar ^\gamma }_1\left(\mathop {\widetilde \prod }\limits_{i{\mathrm{ = 1}}}^2\mu _{i1}^{{{{{\aleph}^{\ell}}_i} \over {\sum\nolimits_{i = 1}^2 {{{\aleph}^{\ell}}_i} }}},\root q \of {1 - \tilde \prod _{i = 1}^{2}{{(1 - \nu _{i1}^{\it q})}^{{{{{\aleph}^{\ell}}_i} \over {\sum\nolimits_{i = 1}^n {{{\aleph}^{\ell}}_i} }}}}} ,\right)   \oplus {\hbar ^\gamma }_2\left(\mathop {\widetilde \prod }\limits_{i{\mathrm{ = 1}}}^2\mu _{i2}^{{{{{\aleph}^{\ell}}_i} \over {\sum\nolimits_{i = 1}^2 {{{\aleph}^{\ell}}_i} }}},\root q \of {1 - \tilde \prod _{i = 1}^{2}{{(1 - \nu _{i2}^{\it q})}^{{{{{\aleph}^{\ell}}_i} \over {\sum\nolimits_{i = 1}^n {{{\aleph}^{\ell}}_i} }}}}} \right)  \cr    &   = {\boldsymbol({\boldsymbol(\mu _{i1}^{{{{{\aleph}^{\ell}}_i} \over {\sum\nolimits_{i = 1}^2 {{{\aleph}^{\ell}}_i} }}}\boldsymbol)^{{\hbar ^\gamma }_1}},\root q \of {1 - \boldsymbol(\tilde \prod _{i = 1}^{2}{{(1 - \nu _{i1}^{\it q})}^{{{{{\aleph}^{\ell}}_i} \over {\sum\nolimits_{i = 1}^n {{{\aleph}^{\ell}}_i} }}}}} \boldsymbol)^{{\hbar ^\gamma }_1}},\boldsymbol)   \oplus {\boldsymbol({\boldsymbol(\mu _{i2}^{{{{{\aleph}^{\ell}}_i} \over {\sum\nolimits_{i = 1}^2 {{{\aleph}^{\ell}}_i} }}}\boldsymbol)^{{\hbar ^\gamma }_2}},\root q \of {1 - \boldsymbol(\tilde \prod _{i = 1}^{2}{{(1 - \nu _{i2}^{\it q})}^{{{{{\aleph}^{\ell}}_i} \over {\sum\nolimits_{i = 1}^n {{{\aleph}^{\ell}}_i} }}}}} \boldsymbol)^{{\hbar ^\gamma }_2}}\boldsymbol)  \cr    &   = {\boldsymbol(\mathop {\widetilde \prod }\limits_{\intercal{\mathrm{ = 1}}}^2{\boldsymbol(\mathop {\widetilde \prod }\limits_{\intercal{\mathrm{ = 1}}}^2\mu _{i{\intercal}}^{{{{{\aleph}^{\ell}}_i} \over {\sum\nolimits_{i = 1}^2 {{{\aleph}^{\ell}}_i} }}}\boldsymbol)^{{\hbar ^\gamma }_{\intercal}}},\root q \of {1 - \tilde \prod _{\intercal = 1}^2\boldsymbol(\tilde \prod _{i = 1}^{2}{{(1 - \nu _{i{\intercal}}^{\it q})}^{{{{{\aleph}^{\ell}}_i} \over {\sum\nolimits_{i = 1}^n {{{\aleph}^{\ell}}_i} }}}}} \boldsymbol)^{{\hbar ^\gamma }_{\intercal}}}\boldsymbol) \cr}$$

The result holds for n = 2 and m = 2.

Suppose that Eq. (1) holds for $n = {k_1}$ and $m = {k_2}$

$$q \!\! -\!\!  ROFSPWG({{\mathcal N}^{\beth}}_{{ {\eth}}_{11}}, \ldots {{\mathcal N}^{\beth}}_{{ {\eth}}{_{{k_1}{k_2}}}}) = \left\langle \mathop{\widetilde \prod}_{\intercal = 1}^{{ k_2}}{\left(\mathop{\widetilde \prod}_{i = 1}^{{ k_1}}\mu _{i{\intercal}}^{{{{{\aleph}^{\it\ell}}_i} \over {\sum\nolimits_{i = 1}^n {{{\aleph}^{\it\ell}}_i} }}}\right)^{{\hbar ^\gamma }_{\intercal}}},\root q \of {1 - \widetilde \prod _{\intercal = 1}^{{k_2}}{{\left(\widetilde \prod _{i = 1}^{{k_1}}{{(1 - \nu _{i{\intercal}}^{\it q})}^{{{{{\aleph}^{\it\ell}}_i} \over {\sum\nolimits_{i = 1}^n {{{\aleph}^{\it\ell}}_i} }}}}\right)}^{{\hbar ^\gamma }_{\intercal}}}} \right\rangle$$

Assume that Eq. (1) holds for $n = {k_1} + 1$ and $= {k_2} + 1$

$$\eqalign{q \!\! -\!\!  ROFSPWG({{\mathcal N}^{\beth}}_{{ {\eth}}_{11}}, &   \ldots {{\mathcal N}^{\beth}}_{{ {\eth}}{_{({k_1} + 1)({k_2} + 1)}}})\\   &  =  \left\{ \oplus _{\intercal = 1}^{{k_2}}{\hbar ^\gamma }_{\intercal}\left( \oplus _{i = 1}^{{k_1}}{{{{\aleph}^{\it\ell}}_i} \over {\sum\nolimits_{i = 1}^n {{{\aleph}^{\it\ell}}_i} }}{{\mathcal N}^{\beth}}_{{ {\eth}}_{i{\intercal}}}\right)\right\} \oplus {\hbar ^\gamma }_{({k_1} + 1)}\left({{{{{\aleph}^{\it\ell}}_i} \over {\sum\nolimits_{i = 1}^n {{{\aleph}^{\it\ell}}_i} }}_{({k_2} + 1)}}{{\mathcal N}^{\beth}}_{{ {\eth}}{_{({k_1} + 1)({k_2} + 1)}}}\right)\\  &  = \left\langle \mathop{\widetilde \prod} _{ \intercal = 1}^{{ k_2}}{\left(\mathop{\widetilde \prod} _{ i = 1}^{{ k_1}}\mu _{i{\intercal}}^{{{{{\aleph}^{\it\ell}}_i} \over {\sum\nolimits_{i = 1}^n {{{\aleph}^{\it\ell}}_i} }}}\right)^{{\hbar ^\gamma }_{\intercal}}},\root q \of {1 - \widetilde \prod _{\intercal = 1}^{{k_2}}{{\left(\widetilde \prod _{i = 1}^{{k_1}}{{(1 - \nu _{i{\intercal}}^{\it q})}^{{{{{\aleph}^{\it\ell}}_i} \over {\sum\nolimits_{i = 1}^n {{{\aleph}^{\it\ell}}_i} }}}}\right)}^{{\hbar ^\gamma }_{\intercal}}}} \right\rangle \\  &  \oplus {\hbar ^\gamma }_{({k_1} + 1)}\left({{{{{\aleph}^{\it\ell}}_i} \over {\sum\nolimits_{i = 1}^n {{{\aleph}^{\it\ell}}_i} }}_{({k_2} + 1)}}{{\mathcal N}^{\beth}}_{{ {\eth}}{_{({k_1} + 1)({k_2} + 1)}}}\right)\\  &  = \left\langle \;\mathop{\widetilde \prod} _{ \intercal = 1}^{ ({k_2} + 1)}{\left(\;\mathop{\widetilde \prod} _{ i = 1}^{ ({k_1} + 1)}\mu _{i{\intercal}}^{{{{{\aleph}^{\it\ell}}_i} \over {\sum\nolimits_{i = 1}^n {{{\aleph}^{\it\ell}}_i} }}}\right)^{{\hbar ^\gamma }_{\intercal}}},\root q \of {1 - \widetilde \prod _{\intercal = 1}^{({k_2} + 1)}{{\left(\widetilde \prod _{i = 1}^{({k_1} + 1)}{{(1 - \nu _{i{\intercal}}^{\it q})}^{{{{{\aleph}^{\it\ell}}_i} \over {\sum\nolimits_{i = 1}^n {{{\aleph}^{\it\ell}}_i} }}}}\right)}^{{\hbar ^\gamma }_{\intercal}}}} \right\rangle}$$

Hence Eq. (1) holds for $n = k1 + 1$ and $m = k2 + 1$, Eq. (1) is true. As a result, Eq. (1) is true for all $m,n \ge 1$ by mathematical induction. Furthermore, to demonstrate that the q-ROFSPWG operator’s aggregated result is actually a q-ROFSN. Now for any ${{\mathcal N}^{\beth}}_{{ {\eth}}_{i{\intercal}}} = ({\nu _{i{\intercal}}},{\mu _{i{\intercal}}}),(i = 1,2,...,\it n)$ and $(\intercal = 1,2,...,\it n)$, where $0 \le {\nu _{i{\intercal}}},\;{\mu _{i{\intercal}}} \le 1,$ satisfying that $0 \le \nu _{i{\intercal}}^{\it q} + \mu _{i{\intercal}}^{\it q} \le 1$ with WVs ${\hbar ^\gamma } = \{ {\hbar ^\gamma }_1,{\hbar ^\gamma }_2, \cdots {\hbar ^\gamma }_n\}$ and ${{{{\aleph}^{\it\ell}}_{\intercal}} \over {\sum\nolimits_{\intercal = 1}^n {{{\aleph}^{\it\ell}}_{\intercal}} }} = \left\{ {{{{{\aleph}^{\it\ell}}_1} \over {\sum\nolimits_{\intercal = 1}^n {{{\aleph}^{\it\ell}}_{\intercal}} }}_1},{{{{{\aleph}^{\it\ell}}_2} \over {\sum\nolimits_{\intercal = 1}^n {{{\aleph}^{\it\ell}}_{\intercal}} }}_2}, \cdots ,{{{{{\aleph}^{\it\ell}}_n} \over {\sum\nolimits_{\intercal = 1}^n {{{\aleph}^{\it\ell}}_{\intercal}} }}_m}\right\}$ for the DMs ${{\mathcal D}_i}$ and for the attributes ${ {\eth}}_{\intercal}^{\prime}$ respectively with the conditions that $\sum\nolimits_{i = 1}^n {{{{{\aleph}^{\it\ell}}_i} \over {\sum\nolimits_{i = 1}^n {{{\aleph}^{\it\ell}}_i} }}} = 1$ and $\sum\nolimits_{\intercal = 1}^m {{\hbar ^\gamma }_{\intercal}} = 1$.

As

$$\eqalign{0 \le {\nu _{i{\intercal}}} \le 1  &  \Rightarrow 0 \le 1 - {\nu _{i{\intercal}}} \le 1 \Rightarrow 0 \le {(1 - \nu _{i{\intercal}}^{\it q})^{{{{{\aleph}^{\it\ell}}_i} \over {\sum\nolimits_{i = 1}^n {{{\aleph}^{\it\ell}}_i} }}}} \le 1\\  &  \Rightarrow 0 \le \mathop{\widetilde \prod} _{ i = 1}^{ n}(1 - \nu _{i{\intercal}}^{\it q}) \le 1 \Rightarrow 0 \le \mathop{\widetilde \prod} _{ \intercal = 1}^{ m}{\left(\mathop{\widetilde \prod} _{ i = 1}^{ n}{(1 - \nu _{i{\intercal}}^{\it q})^{{{{{\aleph}^{\it\ell}}_i} \over {\sum\nolimits_{i = 1}^n {{{\aleph}^{\it\ell}}_i} }}}}\right)^{{\hbar ^\gamma }_{\intercal}}} \le 1 \\  &  \Rightarrow 0 \le \sqrt {\widetilde \prod _{\intercal = 1}^{m}{{\left(\widetilde \prod _{i = 1}^{n}{{(1 - \nu _{i{\intercal}}^{\it q})}^{{{{{\aleph}^{\it\ell}}_i} \over {\sum\nolimits_{i = 1}^n {{{\aleph}^{\it\ell}}_i} }}}}\right)}^{{\hbar ^\gamma }_{\intercal}}}} \le 1}$$

Similarly,

$$\eqalign{0 \le {\mu _{i{\intercal}}} \le 1  &  \Rightarrow 0 \le \mathop{\widetilde \prod} _{ i = 1}^{ n}\mu _{i{\intercal}}^{{{{{\aleph}^{\it\ell}}_i} \over {\sum\nolimits_{i = 1}^n {{{\aleph}^{\it\ell}}_i} }}} \le 1 \\  &  \Rightarrow 0 \le \mathop{\widetilde \prod} _{ \intercal = 1}^{ m}{\left(\mathop{\widetilde \prod} _{ i = 1}^{ n}\mu _{i{\intercal}}^{{{{{\aleph}^{\it\ell}}_i} \over {\sum\nolimits_{i = 1}^n {{{\aleph}^{\it\ell}}_i} }}}\right)^{{\hbar ^\gamma }_{\intercal}}} \le 1\\ \nu _{i{\intercal}}^{\it q} + \mu _{i{\intercal}}^{\it q} \le 1  &  \Rightarrow \mu _{i{\intercal}}^{\it q} \le 1 - \nu _{i{\intercal}}^{\it q} \\  &  \Rightarrow \mathop{\widetilde \prod} _{ i = 1}^{ n}{(\mu _{i{\intercal}}^{\it q})^{{{{{\aleph}^{\it\ell}}_i} \over {\sum\nolimits_{i = 1}^n {{{\aleph}^{\it\ell}}_i} }}}} \le \left(\mathop{\widetilde \prod} _{ i = 1}^{ n}{(1 - \nu _{i{\intercal}}^{\it q})^{{{{{\aleph}^{\it\ell}}_i} \over {\sum\nolimits_{i = 1}^n {{{\aleph}^{\it\ell}}_i} }}}}\right)\\  &  \Rightarrow {\left(\mathop{\widetilde \prod} _{ \intercal = 1}^{ m}{\left(\mathop{\widetilde \prod} _{ i = 1}^{ n}\mu _{i{\intercal}}^{\it q}\right)^{{{{{\aleph}^{\it\ell}}_i} \over {\sum\nolimits_{i = 1}^n {{{\aleph}^{\it\ell}}_i} }}}}\right)^{{\hbar ^\gamma }_{\intercal}}} \le \mathop{\widetilde \prod} _{ \intercal = 1}^{ m}{\left(\mathop{\widetilde \prod} _{ i = 1}^{ n}{(1 - \nu _{i{\intercal}}^{\it q})^{{{{{\aleph}^{\it\ell}}_i} \over {\sum\nolimits_{i = 1}^n {{{\aleph}^{\it\ell}}_i} }}}}\right)^{{\hbar ^\gamma }_{\intercal}}}\\  &  \Rightarrow {\left(\mathop{\widetilde \prod} _{ \intercal = 1}^{ m}{\left(\mathop{\widetilde \prod} _{ i = 1}^{ n}\mu _{i{\intercal}}^{{{{{\aleph}^{\it\ell}}_i} \over {\sum\nolimits_{i = 1}^n {{{\aleph}^{\it\ell}}_i} }}}\right)^{{\hbar ^\gamma }_{\intercal}}}\right)^{\it q}} \le \mathop{\widetilde \prod} _{ \intercal = 1}^{ m}{\boldsymbol(\mathop{\widetilde \prod} _{ i = 1}^{ n}{(1 - \mu _{i{\intercal}}^{\it q})^{{{{{\aleph}^{\it\ell}}_i} \over {\sum\nolimits_{i = 1}^n {{{\aleph}^{\it\ell}}_i} }}}}\boldsymbol)^{{\hbar ^\gamma }_{\intercal}}}}$$

Now we have,

$$0 \le \root q \of {\boldsymbol\{ 1 - \widetilde \prod _{\intercal = 1}^{m}{{\left(\widetilde \prod _{i = 1}^{n}{{(1 - \nu _{i{\intercal}}^{\it q})}^{{{{{\aleph}^{\it\ell}}_i} \over {\sum\nolimits_{i = 1}^n {{{\aleph}^{\it\ell}}_i} }}}}\right)}^{{\hbar ^\gamma }_{\intercal}}}} {\boldsymbol\} ^{\it q}} + {\left\{ \mathop{\widetilde \prod} _{ \intercal = 1}^{ m}{\left(\mathop{\widetilde \prod} _{ i = 1}^{ n}\mu _{i{\intercal}}^{{{{{\aleph}^{\it\ell}}_i} \over {\sum\nolimits_{i = 1}^n {{{\aleph}^{\it\ell}}_i} }}}\right)^{{\hbar ^\gamma }_{\intercal}}}\right\} ^{\it q}}$$

By Eq. (2), we have

$$\le 1 - \mathop{\widetilde \prod} _{ \intercal = 1}^{ m}{\left(\mathop{\widetilde \prod} _{ i = 1}^{ n}{(1 - \nu _{i{\intercal}}^{\it q})^{{{{{\aleph}^{\it\ell}}_i} \over {\sum\nolimits_{i = 1}^n {{{\aleph}^{\it\ell}}_i} }}}}\right)^{{\hbar ^\gamma }_{\intercal}}} + \mathop{\widetilde \prod} _{ \intercal = 1}^{ m}{\left(\mathop{\widetilde \prod} _{ i = 1}^{ n}{(1 - \nu _{i{\intercal}}^{\it q})^{{{{{\aleph}^{\it\ell}}_i} \over {\sum\nolimits_{i = 1}^n {{{\aleph}^{\it\ell}}_i} }}}}\right)^{{\hbar ^\gamma }_{\intercal}}} = 1$$

Therefore,

$$0 \le \root q \of {\boldsymbol\{ 1 - \widetilde \prod _{\intercal = 1}^{m}{{\left(\widetilde \prod _{i = 1}^{n}{{(1 - \nu _{i{\intercal}}^{\it q})}^{{{{{\aleph}^{\it\ell}}_i} \over {\sum\nolimits_{i = 1}^n {{{\aleph}^{\it\ell}}_i} }}}}\right)}^{{\hbar ^\gamma }_{\intercal}}}} {\boldsymbol\} ^{\it q}} + {\left\{ \mathop{\widetilde \prod} _{ \intercal = 1}^{ m}{\left(\mathop{\widetilde \prod} _{ i = 1}^{ n}\mu _{i{\intercal}}^{{{{{\aleph}^{\it\ell}}_i} \over {\sum\nolimits_{i = 1}^n {{{\aleph}^{\it\ell}}_i} }}}\right)^{{\hbar ^\gamma }_{\intercal}}}\right\} ^{\it q}} \le 1$$

**Theorem 4.6** Consider the agglomeration of q-ROFSNs ${{\mathcal N}^{\beth}}_{{ {\eth}}_{i{\intercal}}} = \langle {\mu _{i{\intercal}}},{\nu _{i{\intercal}}}\rangle \;(i = 1,2,...,\it n)$ and $(\intercal = 1,2,...\it m)$ with WVs ${{{{\aleph}^{\it\ell}}_{\intercal}} \over {\sum\nolimits_{\intercal = 1}^n {{{\aleph}^{\it\ell}}_{\intercal}} }} = {\left\{ {{{{{\aleph}^{\it\ell}}_1} \over {\sum\nolimits_{\intercal = 1}^n {{{\aleph}^{\it\ell}}_{\intercal}} }}_1},{{{{{\aleph}^{\it\ell}}_2} \over {\sum\nolimits_{\intercal = 1}^n {{{\aleph}^{\it\ell}}_{\intercal}} }}_2}, \cdots ,{{{{{\aleph}^{\it\ell}}_n} \over {\sum\nolimits_{\intercal = 1}^n {{{\aleph}^{\it\ell}}_{\intercal}} }}_n}\right\} ^T}$ and ${\hbar ^\gamma } = {\{ {\hbar ^\gamma }_1,{\hbar ^\gamma }_2, \cdots {\hbar ^\gamma }_m\} ^T}$ for the DMs ${{\mathcal D}_i}$ and for the attributes ${ {\eth}}_{\intercal}^{\prime}$ respectively with the conditions that $\sum\nolimits_{i = 1}^n {{{{{{{\aleph}^{\it\ell}}_{\intercal}} \over {\sum\nolimits_{\intercal = 1}^n {{{\aleph}^{\it\ell}}_{\intercal}} }}}_i}} = 1$ and $\sum\nolimits_{\intercal = 1}^m {{\hbar ^\gamma }_{\intercal}} = 1.$ Then the q-ROFSPWG operator holds the following characteristics:

(**Idempotency**):

If ${{\mathcal N}^{\beth}}_{{ {\eth}}_{i{\intercal}}} = {\Gamma _e}(\forall i = 1,2,...,n\;{\mathrm{and}}\;\intercal = 1,2,...,\it m)$, where ${\Gamma _e} = (p,r)$, then

$$q \!\! -\!\!  ROFSPWG\left( {{{\mathcal N}^{\beth}}_{11},{{\mathcal N}^{\beth}}_{12}, \ldots {{\mathcal N}^{\beth}}_{nm}} \right) = {\Gamma _e}$$

(**Boundedness**):

If ${{\mathcal N}^{\beth}}_{{ {\eth}}_{i{\intercal}}}^ - = ({\mathrm{mi}}{{\mathrm{n}}_{\intercal}}\;{\mathrm{mi}}{{\mathrm{n}}_i}\{ {\mu _{i{\intercal}}}\} ,{\mathrm{ma}}{{\mathrm{x}}_{\intercal}}\;{\mathrm{ma}}{{\mathrm{x}}_i}\{ {\nu _{i{\intercal}}}\} )$ and ${{\mathcal N}^{\beth}}_{{ {\eth}}_{i{\intercal}}}^ + = ({\mathrm{ma}}{{\mathrm{x}}_{\intercal}}\;{\mathrm{ma}}{{\mathrm{x}}_i}\{ {\mu _{i{\intercal}}}\} ,$ ${\mathrm{mi}}{{\mathrm{n}}_{\intercal}}\;{\mathrm{mi}}{{\mathrm{n}}_i}\{ {\nu _{i{\intercal}}}\} )$, then

$${{\mathcal N}^{\beth}}_{{ {\eth}}_{i{\intercal}}}^ - \le q \!\! -\!\!  ROFSPWG\left( {{{\mathcal N}^{\beth}}_{{ {\eth}}_{11}},{{\mathcal N}^{\beth}}_{{ {\eth}}_{12}},{{\mathcal N}^{\beth}}_{{ {\eth}}_{13}},...,{{\mathcal N}^{\beth}}_{{ {\eth}}_{nm}}} \right) \le {{\mathcal N}^{\beth}}_{{ {\eth}}_{i{\intercal}}}^ +$$

(**Monotonicity**):

If ${\Gamma _{{e_{i{\intercal}}}}} = ({p_{i{\intercal}}},{r_{i{\intercal}}})$, $(\forall i = 1,2,...,n\;{\mathrm{and}}\;\intercal = 1,2,...,\it m)$ be the agglomeration of q-ROFSNs such that ${\mu _{i{\intercal}}} \le {p_{i{\intercal}}}$ and ${\nu _{i{\intercal}}} \ge {r_{i{\intercal}}}$ then

$$q \!\! -\!\!  ROFSPWG\left( {{{\mathcal N}^{\beth}}_{11},{{\mathcal N}^{\beth}}_{12}, \ldots {{\mathcal N}^{\beth}}_{nm}} \right) \le q \!\! -\!\!  ROFSPWG({\Gamma _{11}},{\Gamma _{12}}, \ldots {\Gamma _{nm}})$$

(**Shift Invariance**):

If ${\tilde \Gamma _e} = (p,r)$ is another q-ROFSN, then

$$ q \!\! -\!\!  ROFSPWG\left( {{{\mathcal N}^{\beth}}_{{ {\eth}}_{11}} \oplus {\Gamma _e},{{\mathcal N}^{\beth}}_{{ {\eth}}_{12}} \oplus {\Gamma _e},...{{\mathcal N}^{\beth}}_{{ {\eth}}_{nm}} \oplus {\Gamma _e}} \right) = q \!\! -\!\!  ROFSPWG\left( {{{\mathcal N}^{\beth}}_{{ {\eth}}_{11}},{{\mathcal N}^{\beth}}_{{ {\eth}}_{12}}, \ldots {{\mathcal N}^{\beth}}_{{ {\eth}}_{nm}}} \right) \oplus {\Gamma _e}$$

(**Homogeneity**):

If $\lambda$ is any real number such that $\lambda \ge 0$, then

$$q \!\! -\!\!  ROFSPWG\left( {\lambda {{\mathcal N}^{\beth}}_{{ {\eth}}_{11}},\lambda {{\mathcal N}^{\beth}}_{{ {\eth}}_{12}},...\lambda {{\mathcal N}^{\beth}}_{{ {\eth}}_{nm}}} \right) = \lambda \;q \!\! -\!\!  ROFSPWG\left( {{{\mathcal N}^{\beth}}_{{ {\eth}}_{11}},{{\mathcal N}^{\beth}}_{{ {\eth}}_{12}},...{{\mathcal N}^{\beth}}_{{ {\eth}}_{nm}}} \right)$$

Proofs are straight forward.

## Proposed madm approach

Consider a universal set $U = \{ {u_1},{u_2}, \ldots ,{u_l}\}$ with $l$ alternatives and $E = \{{{ {\eth}}_1},{{ {\eth}}_2}, \ldots ,{{ {\eth}}_n}\}$ be a agglomeration of attributes which contain $n$ elements and ${{ {\eth}}_1} \succ {{ {\eth}}_2} \succ {{ {\eth}}_3} \ldots {{ {\eth}}_n}$ presents the prioritization of attributes which indicates attribute ${ {\eth}}_{\intercal}$ has a higher priority degree than ${{ {\eth}}_i}$ $j \gt i$. If ${\xi ^{\curlyvee}} = \{ {\xi ^{\curlyvee}}_1,{\xi ^{\curlyvee}}_2, \ldots ,{\xi ^{\curlyvee}}_p\}$ is a set of DMs who will evaluate the given ‘m’ alternatives of their respective parameter is a set of decision makers who will valuate the given ‘m’ alternatives of their respective parameters ${ {\eth}}_{\intercal}(\intercal = 1,2,...,\it n)$.

Suppose that the DMs provide their preferences in form of q-ROFSNs ${{\mathcal N}^{\beth}}_{{ {\eth}}_{i{\intercal}}} = ({\mu _{i{\intercal}}},\;{\nu _{i{\intercal}}})$ with the WVs ${{{{\aleph}^{\it\ell}}_{\intercal}} \over {\sum\nolimits_{\intercal = 1}^n {{{\aleph}^{\it\ell}}_{\intercal}} }} = {\left\{ {{{{{\aleph}^{\it\ell}}_1} \over {\sum\nolimits_{\intercal = 1}^n {{{\aleph}^{\it\ell}}_{\intercal}} }}_1},{{{{{\aleph}^{\it\ell}}_2} \over {\sum\nolimits_{\intercal = 1}^n {{{\aleph}^{\it\ell}}_{\intercal}} }}_2}, \cdots ,{{{{{\aleph}^{\it\ell}}_n} \over {\sum\nolimits_{\intercal = 1}^n {{{\aleph}^{\it\ell}}_{\intercal}} }}_n}\right\} ^T}$ and ${\hbar ^\gamma } = {\{ {\hbar ^\gamma }_1,{\hbar ^\gamma }_2, \cdots {\hbar ^\gamma }_m\} ^T}$ for the parameters ${ {\eth}}_{\intercal}^{\prime}$ and decision makers ${\xi ^{\curlyvee}}_i$ respectively with the conditions that $\sum\nolimits_{i = 1}^n {{{{{{{\aleph}^{\it\ell}}_{\intercal}} \over {\sum\nolimits_{\intercal = 1}^n {{{\aleph}^{\it\ell}}_{\intercal}} }}}_i}} = 1$ and $\sum\nolimits_{\intercal = 1}^m {{\hbar ^\gamma }_{\intercal}} = 1.$ The collective data is presented in the decision matrix $M = {[{{\mathcal N}^{\beth}}_{{ {\eth}}_{i{\intercal}}}]_{p \times n}}$. Normalization is not required for the same type of attributes. But there is a possibility of two types of attributes (cost type ${T_c}$ and benefit type ${T_b}$) in MADM process. The decision matrix was then transformed into a normalized matrix $\aleph = {({\aleph_{\it i{\intercal}}})_{\it p \times n}}$, using the normalization procedure,

(5) $${({\aleph_{\it i\intercal}})_{p \times n}} = \left\{ {\matrix{ {{{\mathcal N}^{\beth}}_{{ {\eth}}_{i{\intercal}}};} &  {\intercal \in {\tau _{\it b}}} \cr {{{({{\mathcal N}^{\beth}}_{{ {\eth}}_{i{\intercal}}})}^c};}  &  {\intercal \in {\tau _{\it c}}..} \cr } } \right.$$

where ${({{\mathcal N}^{\beth}}_{{ {\eth}}_{i{\intercal}}})^c}$ represents the compliment of ${{\mathcal N}^{\beth}}_{{ {\eth}}_{i{\intercal}}}$.

We then use the q-ROFSPWA operators or q-ROFSPWG operators to execute a MADM method in a q-ROF situations. The proposed operators will be applied in the MADM and it requires the steps below.

## Case study

Sustainable supplier selection (SSS) is an important aspect of green environment management. It involves identifying and selecting suppliers that share the same commitment to sustainability as the organization, and that can provide environmentally responsible products and services. The importance of sustainable supplier selection in a green environment can be summarized in the following points:

- Reducing environmental impact: Sustainable supplier selection can help to reduce the environmental impact of the supply chain by selecting suppliers that use environmentally responsible practices. This includes suppliers that use renewable energy sources, that minimize waste and pollution, and that have implemented sustainable production processes.
- Meeting customer expectations: As customers become increasingly concerned about the environment, they expect organizations to be environmentally responsible in all aspects of their operations. By selecting sustainable suppliers, organizations can meet these expectations and improve their reputation with customers.
- Mitigating risk: By selecting sustainable suppliers, organizations can mitigate the risk of supply chain disruptions and reputation damage. Suppliers that use environmentally responsible practices are less likely to be subject to regulatory fines and penalties, and are less likely to experience negative publicity due to environmental incidents.
- Improving efficiency: Sustainable supplier selection can also help to improve efficiency in the supply chain. Suppliers that use sustainable practices are often more efficient in their operations, resulting in reduced costs and improved performance.
- Encouraging innovation: By selecting sustainable suppliers, organizations can encourage innovation in environmentally responsible practices. Suppliers that are committed to sustainability are more likely to invest in the research and development of new technologies and processes that reduce their environmental impact.

Organizations should develop a comprehensive supplier selection process that includes sustainability criteria to select sustainable suppliers in a green environment. This process should include evaluating supplier practices and policies related to sustainability, such as energy use, waste management, and carbon emissions. Organizations should also consider supplier certifications and industry ratings related to sustainability. SSS is an important aspect of green environment management. It can help to reduce environmental impact, meet customer expectations, mitigate risk, improve efficiency, and encourage innovation. Organizations should develop a comprehensive supplier selection process that includes sustainability criteria to select suppliers that share their commitment to sustainability and can provide environmentally responsible products and services.

**Algorithm:** 

**Figure 1:**
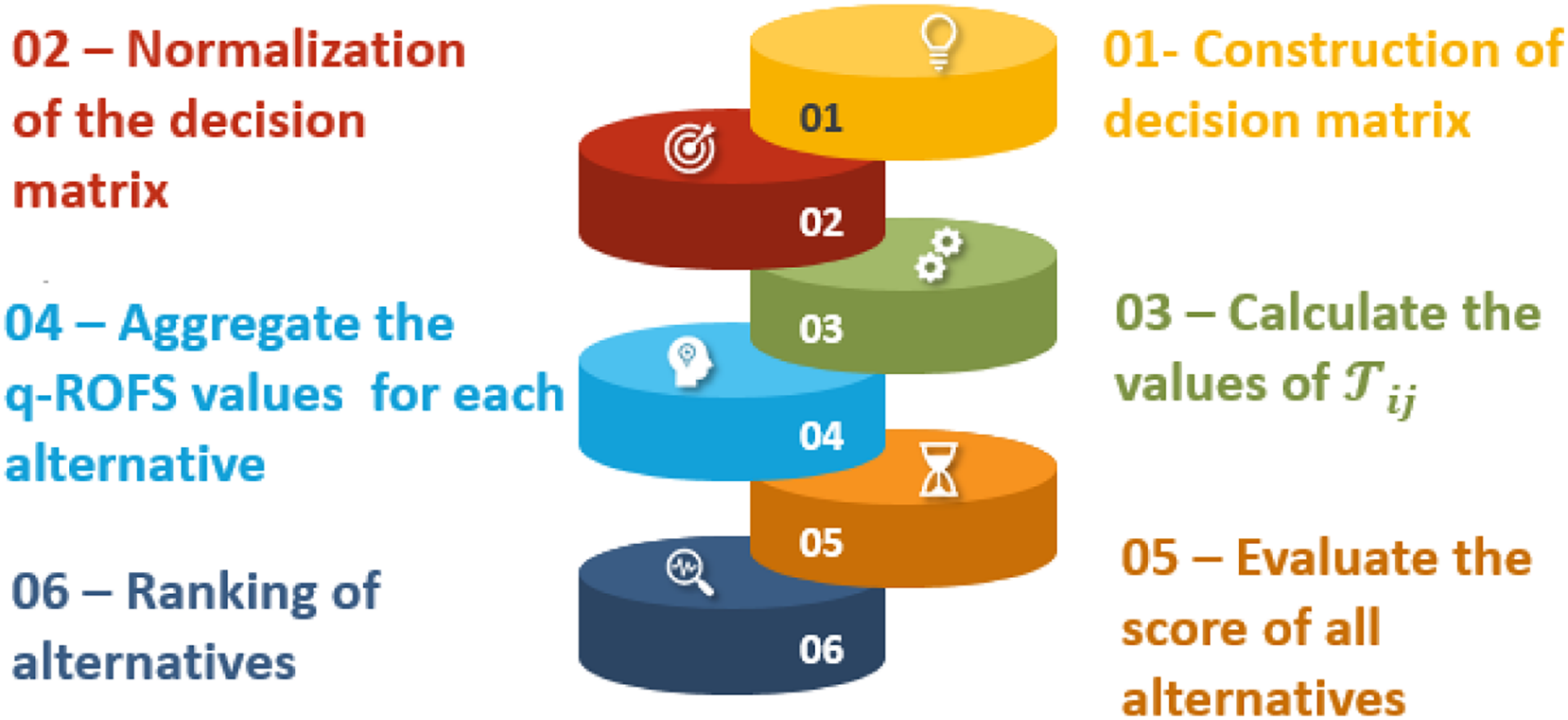
Flow chart of proposed method.

**Input:**
**Step 1:**
Construct a decision matrix $M = {[{{\mathcal N}^{\beth}}_{{ {\eth}}_{i\intercal}}]_{p \times n}}$ by collecting the assessment data of each universal element to their corresponding attributes in the form of q-ROFSNs as:
$$\matrix{ & \eqalign{ {{\xi ^{\curlyvee}}_1} && & {{\xi ^{\curlyvee}}_2} &&&&&&&&&&&&&&&& {} &&&&& {{\xi ^{\curlyvee}}_p}} \cr \eqalign{{{{ {\eth}}_1}} \cr {{{ {\eth}}_2}} \cr {} \cr {{{ {\eth}}_n}} } & \quad {\left[ {\matrix{ {({\mu _{11}},{\nu _{11}})} & {({\mu _{12}},{\nu _{12}})} & { \cdots \cdots } & {({\mu _{1p}},{\nu _{1p}})} \cr {({\mu _{21}},{\nu _{21}})} & {({\mu _{22}},{\nu _{22}}} & { \cdots \cdots } & {({\mu _{2p}},{\nu _{2p}})} \cr \vdots & \vdots & { \ddots \ddots } & \vdots \cr {({\mu _{n1}},{\nu _{n1}})} & {({\mu _{n2}},{\nu _{n2}})} & { \cdots \cdots } & {({\mu _{np}},{\nu _{np}})} } } \right]}} $$
**Step 2:**
There are mainly two different types of attributes are presented in the decision matrix, namely benefit type attribute $({\tau _b})$ and cost type attribute $({\tau _c})$. Normalization is not required for the same type of attributes, but for the different types of attributes in MADM, use the normalization formula given in Eq. (5).
**Calculations:**
**Step 3:**
By using the given formula, calculate the values of ${\breve{\mathcal T}_{i\intercal}}$. By doing so, we are able to calculate the weight vector for all attributes, which is utilized in Step 4. AOs are utilized by this weight veterinarian. Softmax generates its own weight vector, eliminating the need for additional methods to determine weights.
(6) $${\breve {\mathcal T} _{i\intercal}} = \mathop{\widetilde \prod} _{ k = 1}^{ p - 1}{\it H}({{\mathcal N}^{\beth\;}}^{(k)}_{i\intercal})\quad (p = 2 \ldots ,n),$$
$${\breve {\mathcal T} _{i\intercal}} = 1$$
**Step 4:**
By using the q-ROFPWA (or q-ROFPWG) operator, aggregate the values ${\aleph^{\it \ell}}_{i\intercal}$ for each alternative ${{\mathrm{A}}_i}$:
(7) $$q\!\! -\!\! ROFSPWA({{\mathcal N}^{\beth}}_{{ {\eth}}_{11}}, \ldots {{\mathcal N}^{\beth}}_{{ {\eth}}_{nm}}) = \left\langle \root q \of {1 - \widetilde \prod _{\intercal = 1}^m{{\left(\widetilde \prod _{i = 1}^n{{(1 - \mu _{i\intercal}^q)}^{{{{{\aleph}^{\ell}}_{\intercal}} \over {\sum\nolimits_{\intercal = 1}^n {{{\aleph}^{\ell}}_{\intercal}} }}}}\right)}^{{\hbar ^\gamma }_{\intercal}}}} ,\mathop{\widetilde \prod} _{ \intercal = 1}^{ n}{\left(\mathop{\widetilde \prod} _{ \intercal = 1}^{ n}\nu _{i\intercal}^{{{{{\aleph}^{\ell}}_{\intercal}} \over {\sum\nolimits_{\intercal = 1}^n {{{\aleph}^{\ell}}_{\intercal}} }}}\right)^{{\hbar ^\gamma }_{\intercal}}}\right\rangle$$
(8) $$q\!\! -\!\! ROFSPWG({{\mathcal N}^{\beth}}_{{ {\eth}}_{11}}, \ldots {{\mathcal N}^{\beth}}_{{ {\eth}}_{nm}}) = \left\langle \mathop{\widetilde \prod} _{ \intercal = 1}^{ n}{\left(\mathop{\widetilde \prod} _{ \intercal = 1}^{ n}\mu _{i\intercal}^{{{{{\aleph}^{\ell}}_{\intercal}} \over {\sum\nolimits_{\intercal = 1}^n {{{\aleph}^{\ell}}_{\intercal}} }}}\right)^{{\hbar ^\gamma }_{\intercal}}},\root q \of {1 - \widetilde \prod _{\intercal = 1}^m{{\left(\widetilde \prod _{i = 1}^n{{(1 - \nu _{i\intercal}^q)}^{{{{{\aleph}^{\ell}}_{\intercal}} \over {\sum\nolimits_{\intercal = 1}^n {{{\aleph}^{\ell}}_{\intercal}} }}}}\right)}^{{\hbar ^\gamma }_{\intercal}}}} \right\rangle$$
**Output:**
**Step 5:**
Calculate the total score values of each element by using given formula.
(9) $$S(\alpha ) = {{1 + {\mu ^q} - {\nu ^q}} \over 2}$$
**Step 6:**
The alternatives were ranked by the SF, and the best appropriate choice was finally choosen. Pictorial view of proposed method is given in Fig. 1.

The fast growth of economic globalization, along with a more cutthroat climate for business competitiveness, has resulted in the struggle between modern businesses shifting to one that is fought amongst their respective supply chains. The range of individuals who make up the consumer market is expanding, while at the same time, the shelf lives of new products are shrinking. The inconsistency of the demand market and the influence of external factors push businesses toward efficient supply chain implementation and coordination, as well as strategic alliances with other businesses, to strengthen their fundamental competitiveness and protect themselves from the dangers of the outside world. Supplier selection is the most important action to take in order to accomplish this objective. As a result, the challenge of supplier selection has received a significant amount of attention, whether in the context of the theory of supply chain management or the context of practical production management issues.

SSS must consider a wide variety of criteria when operating in an environmentally conscious setting, including environmental performance, social responsibility, economic viability, innovation, transparency and accountability, and supply chain management. When organisations consider these characteristics, they are better able to identify suppliers who are committed to sustainability and more likely to provide sustainable solutions.

A numerical illustration of determining sustainable suppliers by employing q-ROFSNs is provided in this article as a means of illustrating the process that is the subject of this article’s suggested solution. When it comes to supply chain management, there is a panel with four different sustainable suppliers that may be chosen from $U = \{ {\widetilde {\daleth}_1},{\widetilde {\daleth}_2},{\widetilde {\daleth}_3},{\widetilde {\daleth}_4}\}$. The professionals decide to analyze the four potential suppliers based on the following criteria: (1) ${{ {\eth}}_1}$ is the factor for the quality of product; (2) ${{ {\eth}}_2}$ is the factor for the pricing; (3) ${{ {\eth}}_3}$ is the element for the delivery; and (4) ${{ {\eth}}_4}$ is the factor for the ecological concerns.

The application procedure of the proposed approach is as follows:

### Decision-making process

Consider a set $U = \{ {\widetilde {\daleth}^\gamma }_1,{\widetilde {\daleth}^\gamma }_2,{\widetilde {\daleth}^\gamma }_3,{\widetilde {\daleth}^\gamma }_4\}$ of universal elements, a set $E = \{ {{ {\eth}}_1},{{ {\eth}}_2},{{ {\eth}}_3},{{ {\eth}}_4}\}$ of parameters and ${\Phi ^{\beth}} = \{ {\Phi ^{\beth}}_1,{\Phi ^{\beth}}_2,{\Phi ^{\beth}}_3,{\Phi ^{\beth}}_4\}$ be a set of DMs, to assess the universal elements according to their corresponding parameters ${ {\eth}}_{\intercal}\;(\intercal = 1,2,...,\it n)$.

Assume that the DM’s preferences are expressed in q-ROFSNs. ${{\mathcal N}^{\beth}}_{{ {\eth}}_{i{\intercal}}} = ({\mu _{i{\intercal}}},\;{\nu _{i{\intercal}}})$ with the WVs ${{{{\aleph}^{\it\ell}}_{\intercal}} \over {\sum\nolimits_{\intercal = 1}^n {{{\aleph}^{\it\ell}}_{\intercal}} }} = {\left\{ {{{{{\aleph}^{\it\ell}}_1} \over {\sum\nolimits_{\intercal = 1}^n {{{\aleph}^{\it\ell}}_{\intercal}} }}_1},{{{{{\aleph}^{\it\ell}}_2} \over {\sum\nolimits_{\intercal = 1}^n {{{\aleph}^{\it\ell}}_{\intercal}} }}_2}, \cdots ,{{{{{\aleph}^{\it\ell}}_n} \over {\sum\nolimits_{\intercal = 1}^n {{{\aleph}^{\it\ell}}_{\intercal}} }}_n}\right\} ^T}$ and ${\hbar ^\gamma } = {\{ {\hbar ^\gamma }_1,{\hbar ^\gamma }_2, \cdots {\hbar ^\gamma }_m\} ^T}$ for the parameters ${ {\eth}}_{\intercal}^{\prime}$ and decision makers ${\Phi ^{\beth}}_i$ respectively with the conditions that $\sum\nolimits_{i = 1}^n {{{{{{{\aleph}^{\it\ell}}_{\intercal}} \over {\sum\nolimits_{\intercal = 1}^n {{{\aleph}^{\it\ell}}_{\intercal}} }}}_i}} = 1$ and $\sum\nolimits_{\intercal = 1}^m {{\hbar ^\gamma }_{\intercal}} = 1.$ The collective data is presented in the decision matrix $M = {[{{\mathcal N}^{\beth}}_{{ {\eth}}_{i{\intercal}}}]_{p \times n}}$. Normalization is not required for the same type of attributes. But there is a possibility of two types of attributes (cost type ${T_c}$ and benefit type ${T_b}$) in MADM process, the decision matrix then transformed into a normalized matrix $\aleph = {({\aleph_{\it i_{\intercal}}})_{\it p \times n}}$ using normalizing procedure. We take q = 3.

**Step 1:**

From the DMs, obtain a decision matrix ${D^{(p)}} = {(B_{i{\intercal}}^{(p)})_{m \times n}}$ in the form of q-ROFSNs given in Tables 3–6.

**Table 3:** Decision matrix for ${\widetilde {\daleth}^\gamma }_1$.

	${\Phi ^{\beth}}_1$	${\Phi ^{\beth}}_2$	${\Phi ^{\beth}}_3$	${\Phi ^{\beth}}_4$
${{ {\eth}}_1}$	(0.600, 0.140)	(0.110, 0.600)	(0.770, 0.100)	(0.800, 0.400)
${{ {\eth}}_2}$	(0.600, 0.500)	(0.500, 0.020)	(0.010, 0.740)	(0.940, 0.540)
${{ {\eth}}_3}$	(0.430, 0.040)	(0.700, 0.600)	(0.940, 0.130)	(0.570, 0.740)
${{ {\eth}}_4}$	(0.540, 0.900)	(0.710, 0.850)	(0.740, 0.020)	(0.700, 0.070)

**Table 4:** Decision matrix for ${\widetilde {\daleth}^\gamma }_2$.

	${\Phi ^{\beth}}_1$	${\Phi ^{\beth}}_2$	${\Phi ^{\beth}}_3$	${\Phi ^{\beth}}_4$
${{ {\eth}}_1}$	(0.740, 0.530)	(0.510, 0.650)	(0.590, 0.580)	(0.510, 0.610)
${{ {\eth}}_2}$	(0.610, 0.350)	(0.540, 0.710)	(0.610, 0.410)	(0.290, 0.710)
${{ {\eth}}_3}$	(0.530, 0.350)	(0.470, 0.740)	(0.310, 0.570)	(0.910, 0.120)
${{ {\eth}}_4}$	(0.720, 0.290)	(0.820, 0.310)	(0.450, 0.150)	(0.540, 0.140)

**Table 5:** Decision matrix for ${\widetilde {\daleth}^\gamma }_3$.

	${\Phi ^{\beth}}_1$	${\Phi ^{\beth}}_2$	${\Phi ^{\beth}}_3$	${\Phi ^{\beth}}_4$
${{ {\eth}}_1}$	(0.610, 0.250)	(0.100, 0.670)	(0.770, 0.110)	(0.700, 0.170)
${{ {\eth}}_2}$	(0.700, 0.040)	(0.010, 0.740)	(0.800, 0.100)	(0.540, 0.140)
${{ {\eth}}_3}$	(0.650, 0.100)	(0.110, 0.770)	(0.730, 0.110)	(0.750, 0.100)
${{ {\eth}}_4}$	(0.670, 0.110)	(0.140, 0.640)	(0.630, 0.100)	(0.640, 0.140)

**Table 6:** Decision matrix for ${\widetilde {\daleth}^\gamma }_4$.

	${\Phi ^{\beth}}_1$	${\Phi ^{\beth}}_2$	${\Phi ^{\beth}}_3$	${\Phi ^{\beth}}_4$
${{ {\eth}}_1}$	(0.650, 0.110)	(0.640, 0.110)	(0.740, 0.030)	(0.670, 0.100)
${{ {\eth}}_2}$	(0.610, 0.010)	(0.680, 0.070)	(0.500, 0.010)	(0.620, 0.040)
${{ {\eth}}_3}$	(0.710, 0.050)	(0.720, 0.100)	(0.730, 0.020)	(0.710, 0.010)
${{ {\eth}}_4}$	(0.50, 0.160)	(0.500, 0.200)	(0.600, 0.100)	(0.720, 0.020)

**Step 2:**

Normalize the decision matrices acquired by DMs. There is one cost types attribute ${{ {\eth}}_2}$ and others are benefit type attributes given in Table 7–10.

**Table 7:** Normalized decision matrix for ${\widetilde {\daleth}^\gamma }_1$.

	${\Phi ^{\beth}}_1$	${\Phi ^{\beth}}_2$	${\Phi ^{\beth}}_3$	${\Phi ^{\beth}}_4$
${{ {\eth}}_1}$	(0.600, 0.140)	(0.600, 0.110)	(0.770, 0.100)	(0.800, 0.400)
${{ {\eth}}_2}$	(0.600, 0.500)	(0.020, 0.500)	(0.010, 0.740)	(0.940, 0.540)
${{ {\eth}}_3}$	(0.430, 0.040)	(0.600, 0.700)	(0.940, 0.130)	(0.570, 0.740)
${{ {\eth}}_4}$	(0.540, 0.900)	(0.850, 0.710)	(0.740, 0.020)	(0.700, 0.070)

**Table 8:** Normalized decision matrix for ${\widetilde {\daleth}^\gamma }_2$.

	${\Phi ^{\beth}}_1$	${\Phi ^{\beth}}_2$	${\Phi ^{\beth}}_3$	${\Phi ^{\beth}}_4$
${{ {\eth}}_1}$	(0.740, 0.530)	(0.650, 0.510)	(0.590, 0.580)	(0.510, 0.610)
${{ {\eth}}_2}$	(0.610, 0.350)	(0.710, 0.540)	(0.610, 0.410)	(0.290, 0.710)
${{ {\eth}}_3}$	(0.530, 0.350)	(0.740, 0.470)	(0.310, 0.570)	(0.910, 0.120)
${{ {\eth}}_4}$	(0.720, 0.290)	(0.310, 0.820)	(0.450, 0.150)	(0.540, 0.140)

**Table 9:** Normalized decision matrix for ${\widetilde {\daleth}^\gamma }_3$.

	${\Phi ^{\beth}}_1$	${\Phi ^{\beth}}_2$	${\Phi ^{\beth}}_3$	${\Phi ^{\beth}}_4$
${{ {\eth}}_1}$	(0.610, 0.250)	(0.750, 0.570)	(0.450, 0.540)	(0.350, 0.750)
${{ {\eth}}_2}$	(0.570, 0.580)	(0.910, 0.230)	(0.450, 0.710)	(0.350, 0.680)
${{ {\eth}}_3}$	(0.210, 0.590)	(0.350, 0.560)	(0.250, 0.810)	(0.350, 0.650)
${{ {\eth}}_4}$	(0.580, 0.590)	(0.610, 0.210)	(0.350, 0.450)	(0.120, 0.980)

**Table 10:** Normalized decision matrix for ${\widetilde {\daleth}^\gamma }_4$.

	${\Phi ^{\beth}}_1$	${\Phi ^{\beth}}_2$	${\Phi ^{\beth}}_3$	${\Phi ^{\beth}}_4$
${{ {\eth}}_1}$	(0.650, 0.560)	(0.280, 0.910)	(0.350, 0.120)	(0.750, 0.510)
${{ {\eth}}_2}$	(0.350, 0.250)	(0.350, 0.480)	(0.380, 0.490)	(0.590, 0.580)
${{ {\eth}}_3}$	(0.140, 0.940)	(0.380, 0.480)	(0.510, 0.750)	(0.290, 0.340)
${{ {\eth}}_4}$	(0.480, 0.910)	(0.250, 0.450)	(0.710, 0.290)	(0.390, 0.210)

**Step 3:**

Compute the values of $\breve {T}_ {i{\intercal}} ^{(p)}$ by using Equation $\intercal {\rm ti}\intercal3$.

$$\breve {T} _{i{\intercal}}^{(1)} = \left( {\matrix{ 1  &  1  &  1  &  1 \cr {0.6060}  &  {0.6070}  &  {0.7270}  &  {0.7240} \cr {0.3300}  &  {0.2650}  &  {0.2160}  &  {0.6050} \cr {0.1780}  &  {0.1150}  &  {0.1970}  &  {0.2350} \cr } } \right)$$

We obtain the WVs

$$\eqalign{{W_1} &  = \left( {0.4730,\;0.2860,\;0.1560,\;0.0840} \right)\\{W_2}   &  =  (0.5030,\;0.3050,\;0.1330,\;0.0570)\\{W_3}   &  =  (0.4670,\;0.3390,\;0.1000,\;0.0920)\\{W_4}   &  =  (0.3900,\;0.2820,\;0.2350,\;0.0910)}$$

$$\breve {T} _{i{\intercal}}^{(2)} = \left( {\matrix{ 1  &  1  &  1  &  1 \cr {0.6280}  &  {0.5700}  &  {0.5050}  &  {0.4520} \cr {0.3710}  &  {0.3420}  &  {0.2920}  &  {0.1500} \cr {0.2050}  &  {0.0760}  &  {0.1230}  &  {0.1310} \cr } } \right)$$

We obtain the WVs

$$\eqalign{{W_1}   &  =  (0.4530,\;0.2840,\;0.1680,\;0.0930)\\ {W_2}   &  =  (0.5030,\;0.2860,\;0.1720,\;0.0380)\\ {W_3}   &  =  (0.5200,\;0.2630,\;0.1520,\;0.0640)\\ {W_4}   &  =  (0.5770,\;0.2600,\;0.0860,\;0.0750)}$$

$$\breve {T} _{i{\intercal}}^{(3)} = \left( {\matrix{ 1  &  1  &  1  &  1 \cr {0.6050}  &  {0.6180}  &  {0.4660}  &  {0.3100} \cr {0.2420}  &  {0.5370}  &  {0.1700}  &  {0.1120} \cr {0.1200}  &  {0.2320}  &  {0.0410}  &  {0.0430} \cr } } \right)$$

We obtain the WVs

$$\eqalign{{W_1}   &  =  (0.5080,\;0.3070,\;0.1420,\;0.0610)\\ {W_2}   &  =  (0.4180,\;0.2580,\;0.2240,\;0.0970)\\{W_3}   &  =  (0.5960,\;0.2770,\;0.1010,\;0.0240)\\ {W_4}   &  =  (0.6820,\;0.2110,\;0.0760,\;0.0290)}$$

$$\breve {T} _{i{\intercal}}^{(1)} = \left( {\matrix{ 1  &  1  &  1  &  1 \cr {0.6060}  &  {0.6070}  &  {0.7270}  &  {0.7240} \cr {0.3300}  &  {0.2650}  &  {0.2160}  &  {0.6050} \cr {0.1780}  &  {0.1150}  &  {0.1970}  &  {0.2350} \cr } } \right)$$

We obtain the WVs

$$\eqalign{{W_1} &  = (0.4650,\;0.2550,\;0.2380,\;0.4000)\\{W_2}   &  =  (0.4820,\;0.0640,\;0.2240,\;0.2270)\\{W_3}   &  =  (0.4260,\;0.2210,\;0.1990,\;0.1510)\\{W_4}   &  =  (0.3780,\;0.2430,\;0.1910,\;0.1860)}$$

**Step 4:**

Calculate the aggregated values ${\aleph^{\ell}}_i$ for each alternative ${\widetilde {\daleth}^\gamma }_i$ by using the q-ROFSPWA and q-ROFSPWG operators using Eqs. (7), (8), respectively given in Table 11.

**Table 11:** q-ROFS aggregated values.

	q-ROFSPWA	q-ROFSPWG
${\widetilde {\daleth}^\gamma }_1$	(0.6954, 0.2176)	(0.3721, 0.4576)
${\widetilde {\daleth}^\gamma }_2$	(0.6664, 0.4812)	(0.2136, 0.5703)
${\widetilde {\daleth}^\gamma }_3$	(0.6171, 0.4983)	(0.3410, 0.2314)
${\widetilde {\daleth}^\gamma }_4$	(0.5614, 0.0000)	(0.1456, 0.0027)
${\widetilde {\daleth}^\gamma }_5$	(0.4946, 0.4160)	(0.6112, 0.1288)

**Step 5:**

By using Eq. (9), calculate the score of all q-ROFS aggregated values ${\aleph^{\ell}}_i$, given in Table 12.

**Table 12:** Score of q-ROFS aggregated values.

${\mathcal S}({\widetilde {\daleth}^\gamma }_i)$	$q\!\! -\!\! ROFSPWA$	$q\!\! -\!\! ROFSPWG$
${\mathcal S}({\widetilde {\daleth}^\gamma }_1)$	0.8648	0.3304
${\mathcal S}({\widetilde {\daleth}^\gamma }_2)$	0.8611	0.3044
${\mathcal S}({\widetilde {\daleth}^\gamma }_3)$	0.8600	0.3048
${\mathcal S}({\widetilde {\daleth}^\gamma }_4)$	0.6472	0.2988

**Step 6:**

As per given in Table 12, the alternative ${\widetilde {\daleth}^\gamma }_1$ has the maximum score. So, ${\widetilde {\daleth}^\gamma }_1$ is the optimal solution,

### Authenticity analysis

Wang & Triantaphyllou (2008) evaluated the following test criteria to demonstrate the validity of the suggested approach.

- The optimum alternative should not change if the ratings of the non-optimal universal elements are replaced with those of the worse option, provided that the corresponding WVs remains persistent.
- The whole framework of technique should be transitive.
- When the same MADM approach is applied to solve the problem, the aggregated result of the alternatives should be identical as the assessment of the initial problem.

We confirmed the conditions on our proposed MADM approach in the part below.

#### Authenticity test 1

If the PMVs and NMVs of alternatives ${\widetilde {\daleth}^\gamma }_2$ and ${\widetilde {\daleth}^\gamma }_3$ in Tables 4 and 5 are exchanged, the modified decision matrix given in Tables 13 and 14 appears.

**Table 13:** Modified decision matrix for ${\widetilde {\daleth}^\gamma }_2$.

	${\Phi ^{\beth}}_1$	${\Phi ^{\beth}}_2$	${\Phi ^{\beth}}_3$	${\Phi ^{\beth}}_4$
${{ {\eth}}_1}$	(0.53, 0.74)	(0.51, 0.65)	(0.85, 0.95)	(0.51, 0.61)
${{ {\eth}}_2}$	(0.35, 0.61)	(0.54, 0.71)	(0.61, 0.41)	(0.29, 0.71)
${{ {\eth}}_3}$	(0.35, 0.53)	(0.47, 0.74)	(0.57, 0.31)	(0.12, 0.91)
${{ {\eth}}_4}$	(0.72, 0.29)	(0.82, 0.31)	(0.15, 0.45)	(0.14, 0.54)

**Table 14:** Modified decision matrix for ${\widetilde {\daleth}^\gamma }_3$.

	${\Phi ^{\beth}}_1$	${\Phi ^{\beth}}_2$	${\Phi ^{\beth}}_3$	${\Phi ^{\beth}}_4$
${{ {\eth}}_1}$	(0.25, 0.61)	(0.57, 0.75)	(0.54, 0.45)	(0.75, 0.35)
${{ {\eth}}_2}$	(0.58, 0.57)	(0.23, 0.91)	(0.71, 0.45)	(0.68, 0.35)
${{ {\eth}}_3}$	(0.59, 0.21)	(0.56, 0.35)	(0.81, 0.25)	(0.65, 0.35)
${{ {\eth}}_4}$	(0.59, 0.58)	(0.21, 0.61)	(0.45, 0.35)	(0.98, 0.12)

On the basis of given data, the proposed q-ROFSPWA operator has been applied and aggregate the q-ROFSNs for the alternatives, given in Table 15.

**Table 15:** Collective q-ROF decision matrix.

${\mathcal S}({\aleph^{\ell}}_i)$	$q\!\! -\!\! ROFSPWA$	$q\!\! -\!\! ROFSPWG$
${\mathcal S}({\aleph^{\ell}}_1)$	0.6749	0.2314
${\mathcal S}({\aleph^{\ell}}_2)$	0.5453	0.0871
${\mathcal S}({\aleph^{\ell}}_3)$	0.2312	0.3212
${\mathcal S}({\aleph^{\ell}}_4)$	0.2910	0.2912

As a result, the ranking order of options is determined by the score values, which is same as the original ranking and proposed technique passes the first three tests.

#### Authenticity test 2, test 3

If we split the given problem like $\{ {\widetilde {\daleth}^\gamma }_1,{\widetilde {\daleth}^\gamma }_2\} ,\;\{ {\widetilde {\daleth}^\gamma }_2,{\widetilde {\daleth}^\gamma }_3\} ,\;\{ {\widetilde {\daleth}^\gamma }_3,{\widetilde {\daleth}^\gamma }_4\} ,\;\{ {\widetilde {\daleth}^\gamma }_4,{\widetilde {\daleth}^\gamma }_1\}$. Then using the proposed method, we obtain the following ranking order ${\widetilde {\daleth}^\gamma }_1 \ge {\widetilde {\daleth}^\gamma }_2,\;{\widetilde {\daleth}^\gamma }_2 \ge {\widetilde {\daleth}^\gamma }_3,\;{\widetilde {\daleth}^\gamma }_4 \ge {\widetilde {\daleth}^\gamma }_3,\;{\widetilde {\daleth}^\gamma }_1 \ge {\widetilde {\daleth}^\gamma }_4$ and this is identical to the original ranking. As a consequence, the proposed approach satisfies authenticity test 2 and test 3.

### Sensitivity analysis

Across the whole decision-making process, the effect of q on the most preferable decision was studied, utilizing multiple values of q for the given scenario. Table 16 epitomizes the total score values and ranking of the alternatives associated to these distinct q values. Since the impact of ‘q’ on decision-making, our proposed technique is more adaptable because the DMs may adjust the attributes based on their current circumstances and preferences. For example, if the DMs can draw inferences from their optimistic tendency, low values may be given to these criteria and the overall score values will decrease. If the DMs are optimistic, the parameters can be given higher values and the aggregated values of scores will increase. As a consequence, the results are accurate. The DM might be able to determine their goals *via* this analysis and pick the optimal option depending on their perspective. The optimal alternative is identical, implying that the results are accurate and affected by the DM’s optimism. The results of the rating are valid. The DM may be able to see their objectives *via* this analysis and pick the optimal option depending on their perspective. The optimal alternative is the same, implying that the results are factual and affected by the DM’s optimism. The results of the rating are valid. The DM may be able to see their objectives *via* this analysis and pick the optimal option depending on their perspective.

**Table 16:** Final ranking.

q	${\mathcal S}({\widetilde {\daleth}^\gamma }_1)$	${\mathcal S}({\widetilde {\daleth}^\gamma }_2)$	${\mathcal S}({\widetilde {\daleth}^\gamma }_3)$	${\mathcal S}({\widetilde {\daleth}^\gamma }_4)$	Ranking order	Final decision
4	0.6058	0.4363	0.3554	0.1331	${\tilde {\daleth}^\gamma }_1 \succ {\tilde {\daleth}^\gamma }_2 \succ {\tilde {\daleth}^\gamma }_3 \succ {\tilde {\daleth}^\gamma }_4$	${\tilde {\daleth}^\gamma }_1$
5	0.2211	0.1130	0.2140	0.1202	${\tilde {\daleth}^\gamma }_1 \succ {\tilde {\daleth}^\gamma }_3 \succ {\tilde {\daleth}^\gamma }_4 \succ {\tilde {\daleth}^\gamma }_2$	${\tilde {\daleth}^\gamma }_1$
6	0.5126	0.4160	0.0361	0.3062	${\tilde {\daleth}^\gamma }_1 \succ {\tilde {\daleth}^\gamma }_2 \succ {\tilde {\daleth}^\gamma }_4 \succ {\tilde {\daleth}^\gamma }_3$	${\tilde {\daleth}^\gamma }_1$

### Comparison analysis and discussion

In this section, we will analyze and compare the new operators we are suggesting with the operators that are already being used. The important point to note is that both our proposed operators and the existing ones lead to the same conclusion. This demonstrates that our suggested operators are superior.

To understand this, we conducted an investigation and found that by using specific preexisting operators to process the information, we can reach an equally optimal conclusion. This highlights the strength and reliability of our proposed approach, which enables us to make perfect decisions. To provide a clear comparison, we have included Table 17, which shows how our suggested operators compare to the various existing operators that are currently in use.

**Table 17:** Comparison of proposed operators with some exiting operators.

Authors	AOs	Ranking of alternatives	The optimal alternative
Peng, Dai & Garg (2018)	q-ROFWEA	${\widetilde {\daleth}^\gamma }_1 \succ {\widetilde {\daleth}^\gamma }_3 \succ {\widetilde {\daleth}^\gamma }_4 \succ {\widetilde {\daleth}^\gamma }_2$	${\widetilde {\daleth}^\gamma }_1$
	q-ROFDWG	${\widetilde {\daleth}^\gamma }_1 \succ {\widetilde {\daleth}^\gamma }_3 \succ {\widetilde {\daleth}^\gamma }_2 \succ {\widetilde {\daleth}^\gamma }_4$	${\widetilde {\daleth}^\gamma }_1$
Liu & Wang (2018)	q-ROFWA	${\widetilde {\daleth}^\gamma }_1 \succ {\widetilde {\daleth}^\gamma }_2 \succ {\widetilde {\daleth}^\gamma }_3 \succ {\widetilde {\daleth}^\gamma }_4$	${\widetilde {\daleth}^\gamma }_1$
	q-ROFWG	${\widetilde {\daleth}^\gamma }_1 \succ {\widetilde {\daleth}^\gamma }_2 \succ {\widetilde {\daleth}^\gamma }_4 \succ {\widetilde {\daleth}^\gamma }_3$	${\widetilde {\daleth}^\gamma }_1$
Riaz et al. (2020)	q-ROFHWAGA	${\widetilde {\daleth}^\gamma }_1 \succ {\widetilde {\daleth}^\gamma }_2 \succ {\widetilde {\daleth}^\gamma }_3 \succ {\widetilde {\daleth}^\gamma }_4$	${\widetilde {\daleth}^\gamma }_1$
	q-ROFHOWAGA	${\widetilde {\daleth}^\gamma }_1 \succ {\widetilde {\daleth}^\gamma }_3 \succ {\widetilde {\daleth}^\gamma }_2 \succ {\widetilde {\daleth}^\gamma }_4$	${\widetilde {\daleth}^\gamma }_1$
Liu, Wang & Liu (2018)	q-ROFHM	${\widetilde {\daleth}^\gamma }_1 \succ {\widetilde {\daleth}^\gamma }_2 \succ {\widetilde {\daleth}^\gamma }_3 \succ {\widetilde {\daleth}^\gamma }_4$	${\widetilde {\daleth}^\gamma }_1$
	q-ROFWHM	${\widetilde {\daleth}^\gamma }_1 \succ {\widetilde {\daleth}^\gamma }_2 \succ {\widetilde {\daleth}^\gamma }_3 \succ {\widetilde {\daleth}^\gamma }_4$	${\widetilde {\daleth}^\gamma }_1$
Riaz et al. (2020)	q-ROFEPWA	${\widetilde {\daleth}^\gamma }_1 \succ {\widetilde {\daleth}^\gamma }_2 \succ {\widetilde {\daleth}^\gamma }_3 \succ {\widetilde {\daleth}^\gamma }_4$	${\widetilde {\daleth}^\gamma }_1$
Zhao et al. (2010)	q-ROFHM	${\widetilde {\daleth}^\gamma }_1 \succ {\widetilde {\daleth}^\gamma }_2 \succ {\widetilde {\daleth}^\gamma }_4 \succ {\widetilde {\daleth}^\gamma }_3$	${\widetilde {\daleth}^\gamma }_1$
	q-ROFWHM	${\widetilde {\daleth}^\gamma }_1 \succ {\widetilde {\daleth}^\gamma }_2 \succ {\widetilde {\daleth}^\gamma }_3 \succ {\widetilde {\daleth}^\gamma }_4$	${\widetilde {\daleth}^\gamma }_1$
	q-ROFEPWG	${\widetilde {\daleth}^\gamma }_1 \succ {\widetilde {\daleth}^\gamma }_4 \succ {\widetilde {\daleth}^\gamma }_3 \succ {\widetilde {\daleth}^\gamma }_2$	${\widetilde {\daleth}^\gamma }_1$
Joshi & Gegov (2020)	CQROFWA	${\widetilde {\daleth}^\gamma }_1 \succ {\widetilde {\daleth}^\gamma }_2 \succ {\widetilde {\daleth}^\gamma }_3 \succ {\widetilde {\daleth}^\gamma }_4$	${\widetilde {\daleth}^\gamma }_1$
	CQROFWG	${\widetilde {\daleth}^\gamma }_1 \succ {\widetilde {\daleth}^\gamma }_2 \succ {\widetilde {\daleth}^\gamma }_3 \succ {\widetilde {\daleth}^\gamma }_4$	${\widetilde {\daleth}^\gamma }_1$
Liu & Liu (2018)	q-ROFWBM	${\widetilde {\daleth}^\gamma }_1 \succ {\widetilde {\daleth}^\gamma }_2 \succ {\widetilde {\daleth}^\gamma }_3 \succ {\widetilde {\daleth}^\gamma }_4$	${\widetilde {\daleth}^\gamma }_1$
	q-ROFWGBM	${\widetilde {\daleth}^\gamma }_1 \succ {\widetilde {\daleth}^\gamma }_3 \succ {\widetilde {\daleth}^\gamma }_2 \succ {\widetilde {\daleth}^\gamma }_4$	${\widetilde {\daleth}^\gamma }_1$
Jana, Muhiuddin & Pal (2019)	q-ROFDWA	${\widetilde {\daleth}^\gamma }_1 \succ {\widetilde {\daleth}^\gamma }_2 \succ {\widetilde {\daleth}^\gamma }_3 \succ {\widetilde {\daleth}^\gamma }_4$	${\widetilde {\daleth}^\gamma }_1$
Proposed	q-ROFSPWA	${\widetilde {\daleth}^\gamma }_1 \succ {\widetilde {\daleth}^\gamma }_2 \succ {\widetilde {\daleth}^\gamma }_3 \succ {\widetilde {\daleth}^\gamma }_4$	${\widetilde {\daleth}^\gamma }_1$
	q-ROFSPWG	${\widetilde {\daleth}^\gamma }_1 \succ {\widetilde {\daleth}^\gamma }_3 \succ {\widetilde {\daleth}^\gamma }_2 \succ {\widetilde {\daleth}^\gamma }_4$	${\widetilde {\daleth}^\gamma }_1$

## Conclusion

Under the parameterized description of the universe’s elements, the q-ROFSSs are more efficient because they provide a broad range for PMV and NMV to cope with ambiguous and imprecise data. AOs are essential mathematical instruments for information fusion, which is the process of reducing a collection of fuzzy numbers to a single fuzzy number that represents the set uniquely. We developed two distinct AOs for data fusion of q-ROFSNs in order to surmount some of the drawbacks of existing AOs. Based on prescribed operational laws, we developed the q-ROFSPWA operator and q-ROFSPWG operator AOs. We also presented a robust MADM strategy to demonstrate the efficacy and superiority of the proposed AOs. A numerical example of the proposed MADM technique in relation to the problem of selecting a sustainable logistic provider is also provided to illustrate the uncertain condition. The outcomes demonstrate that the proposed method for addressing uncertainty is both precise and efficient. In order to demonstrate the efficacy of proposed AOs, we discuss authenticity analysis. Finally, the efficacy, precision, and veracity of the proposed AOs are assessed by comparing the proposed MADM technique to various previous approaches.

Regarding the limits of our proposed work, there is no inclusion of the interplay between membership and non-membership recommended by the DMs, and if our data is not q-ROFNs, it will not function effectively. The suggested model functions effectively with q-ROFNs as input. However, with some small modifications, the suggested model may be expanded to include more input data types. Future research will examine how the proposed operators may be used for various forms of data and how they function in various domains. The principles in this article can be applied to a wide range of real-world situations. Effectively addressing ambiguity in business, machine intelligence, cognitive science, the electoral system, pattern recognition, learning techniques, trade analysis, predictions, agricultural estimate, microelectronics, and other fields is possible with the help of these methods.

## Additional Information and Declarations

### Data Availability

The following information was supplied regarding data availability: Raw data is available in Tables 3–6.
